# Interleukin-5 (IL-5) Therapy Prevents Allograft Rejection by Promoting CD4^+^CD25^+^ Ts2 Regulatory Cells That Are Antigen-Specific and Express IL-5 Receptor

**DOI:** 10.3389/fimmu.2021.714838

**Published:** 2021-11-29

**Authors:** Bruce M. Hall, Rachael M. Hall, Giang T. Tran, Catherine M. Robinson, Paul L. Wilcox, Prateek K. Rakesh, Chuanmin Wang, Alexandra F. Sharland, Nirupama D. Verma, Suzanne J. Hodgkinson

**Affiliations:** ^1^ Immune Tolerance Laboratory, South West Clinical School, University of New South Wales (UNSW) Sydney, Liverpool, NSW, Australia; ^2^ Ingham Institute of Applied Medical Research, Liverpool Hospital, Liverpool, NSW, Australia; ^3^ Transplantation Immunobiology Group, Central Clinical School, Faculty of Medicine and Health, The University of Sydney, Sydney, NSW, Australia

**Keywords:** interleukin-5, transplant tolerance, T regulatory cells, cytokines, allograft rejection, chronic rejection, CD4 + CD25 + Treg cells, Th2 cytokines

## Abstract

CD4^+^CD25^+^Foxp3^+^T cell population is heterogenous and contains three major sub-groups. First, thymus derived T regulatory cells (tTreg) that are naïve/resting. Second, activated/memory Treg that are produced by activation of tTreg by antigen and cytokines. Third, effector lineage CD4^+^CD25^+^T cells generated from CD4^+^CD25^-^ T cells’ activation by antigen to transiently express CD25 and Foxp3. We have shown that freshly isolated CD4^+^CD25^+^T cells are activated by specific alloantigen and IL-4, not IL-2, to Ts2 cells that express the IL-5 receptor alpha. Ts2 cells are more potent than naïve/resting tTreg in suppressing specific alloimmunity. Here, we showed rIL-5 promoted further activation of Ts2 cells to Th2-like Treg, that expressed *foxp3, irf4, gata3* and *il5. In vivo*, we studied the effects of rIL-5 treatment on Lewis heart allograft survival in F344 rats. Host CD4^+^CD25^+^T cells were assessed by FACS, in mixed lymphocyte culture and by RT-PCR to examine mRNA of Ts2 or Th2-like Treg markers. rIL-5 treatment given 7 days after transplantation reduced the severity of rejection and all grafts survived ≥60d whereas sham treated rats fully rejected by day 31 (p<0.01). Treatment with anti-CD25 or anti-IL-4 monoclonal antibody abolished the benefits of treatment with rIL-5 and accelerated rejection. After 10d treatment with rIL-5, hosts’ CD4^+^CD25^+^ cells expressed more *Il5ra* and responded to specific donor Lewis but not self. Enriched CD4^+^CD25^+^ cells from rIL-5 treated rats with allografts surviving >60 days proliferated to specific donor only when rIL-5 was present and did not proliferate to self or third party. These cells had more mRNA for molecules expressed by Th2-like Treg includin*g Irf4, gata3* and *Il5.* These findings were consistent with IL-5 treatment preventing rejection by activation of Ts2 cells and Th2-like Treg.

## Introduction

With current immunosuppression, organ allografts are rarely lost from acute rejection but later rejection remains a major problem (1) in all forms of organ transplantation. No current therapy is effective at its prevention or treatment (2–4). Induction of alloantigen specific tolerance is a potential therapy to prolong graft survival.

Rejection is a complex immunological process, starting with CD4^+^T cell activation by donor alloantigen (4, 5) resulting in a mononuclear cell infiltrate, T cell mediated injury (6, 7) and antibody deposition (8) with activation of complement (9). This leads to slow destruction of the allograft from vascular injury (10), destruction of the microcirculation (11, 12) and fibrosis (1).

The most frequently studied Treg are naïve/resting thymus derived CD4^+^CD25^+^Foxp3^+^T cells (tTreg) (13, 14) however these alone do not mediate transplant tolerance. Transplanted tissues, while activating rejection responses, also induce alloantigen-specific CD4^+^CD25^+^Foxp3^+^Treg (15–17).

CD4^+^CD25^+^Foxp3^+^T cell population is heterogenous, containing three major sub-groups, as described by Miyara et al. (18). Understanding this heterogeneity may be useful in activating Treg as therapy (19–21), an approach that is yet to fully evolve, as reviewed (22). In **Table 1** we define the subsets of CD4^+^CD25^+^Foxp3^+^T cells, relevant to the understanding of this work.

**Table 1:** Definition of subpopulations of cells within peripheral lymphoid CD4^+^CD25^+^Foxp3^+^T cells relevant to this study.

**Thymic derived naive/resting Treg.**	Thymus derived CD4^+^CD25^+^Foxp3^+^T cells that have not been activated by antigen since leaving the thymus. Known as tTreg or nTreg. These cells are the majority of CD4^+^CD25^+^T cells in naive animals.
**Activated/memory Treg**	Thymus derived CD4^+^CD25^+^ Foxp3^+^Treg that are activated by antigen in the periphery in the presence of cytokines which induces higher expression of CD25 and Foxp3 than in tTreg
**Peripheral/induced Treg**	Effector lineage CD4^+^CD25^-^Foxp3^-^T cells that have been activated by specific antigen in the absence of inflamatory cytokines such as IL-6 and IL-1 and transiently express CD25 and Foxp3. Known as pTreg or iTreg
**Ts1 cells**	tTreg that have been activated by a specific antigen and the Type-1 cytokine IL-2. Express receptors for Type-1 cytokines IFNGR and IL-12Rβ2. Ts1 cells are 10-64 times more potent than tTreg at suppression of responses to specific-antigen.
**Th1-like Treg**	Ts1 cells that have been activated by a specific-antigen and the Type-1 cytokines such as IL-12 or IFN-γ. IL-2 blocks induction of Th1-like Treg. Also express Th1 associated molecules IFN-γ, Tbet, CXCR3. Th1-like Treg’s suppression is 100-1000 fold more potent than tTreg.
**Ts2 cells**	tTreg that have been activated by a specific antigen and the Type-2 cytokine IL-4. IL-2 is not required for induction of Ts2 cells as tTreg express IL-4Rα. Ts2 cells express receptors for Type-2 cytokine IL-5 and 10-32 times more potent at suppression of responses to specific antigen than tTreg.
**Th2-like Treg**	Ts2 cells that have been activated by a specific-antigen and the Type-2 cytokine IL-5. Also express Th2 associated molecules IL-5, GATA3, IRF4 and CCR8. Suppression 100-1000 fold more potent than tTreg

Within peripheral CD4^+^CD25^+^ cells in addition to naïve/resting tTreg there are tTreg that have been activated by antigen and cytokines known as activated/memory Treg, and effector lineage CD4^+^CD25^+^T cells that have been activated in periphery by antigen and transiently express CD25 and Foxp3, also known as pTreg/iTreg. Thus, the enriched CD4^+^CD25^+^ cells we study contain all three populations and have a vast array of T cell receptors that can each react to a specific antigen.

In animals that develop transplant tolerance, control of rejection is mainly mediated by alloantigen-specific CD4^+^CD25^+^Foxp3^+^Treg (15–17, 23). In these animals, antigen-specific Treg are expanded. Thus, promotion of alloantigen-specific Treg could control rejection and establish operational tolerance (24).

Treg, either freshly isolated or after polyclonal expansion, need to be at ratios of ≥1:1 to effector T cells to fully suppress immune responses (14, 25) including allograft rejection *in vivo* (17) and proliferation *in vitro* of naïve CD4^+^T cells to alloantigen in mixed lymphocyte culture (MLC) (26). However, *in vivo* the ratio of Treg (CD4^+^CD25^+^Foxp3^+^T cells) to effector T cells (CD4^+^CD25^-^Foxp3^-^) is highly regulated to ≤1:10 and ratios of 1:1 cannot be maintained.

The CD4^+^CD25^+^Foxp3^+^Treg that are antigen activated and mediate alloantigen-specific tolerance are also present within the CD4^+^CD25^+^T cell pool. They are more potent at suppression, and have different properties and phenotypes to naïve/resting CD4^+^CD25^+^Foxp3^+^Treg (27). Preparations of CD4^+^CD25^+^cells contain both naïve resting tTreg and activated antigen-specific Treg.

The precise pathway for activation of alloantigen specific CD4^+^CD25^+^Foxp3^+^Treg are still not known, however. We have previously shown activation of naïve/resting Treg with specific-alloantigen and the Type-1 cytokine IL-2 induces a population of more potent antigen-specific Treg that express *Ifngr* and *Il12rb2* (28, 29). We have called these cells Ts1. Ts1 cells are promoted by alloantigen and the Th1 cytokines IFN-γ and/or IL-12 to Th1-like Treg that suppress at very low ratios (23) and can induce transplant tolerance.

In a rejection response, there is also activation of Th2, Th17 and other cell types that produce different cytokines to Th1 cells. These different cytokines in presence of alloantigen also promote activation of naïve Treg by separate pathways.

**Figure 1:**
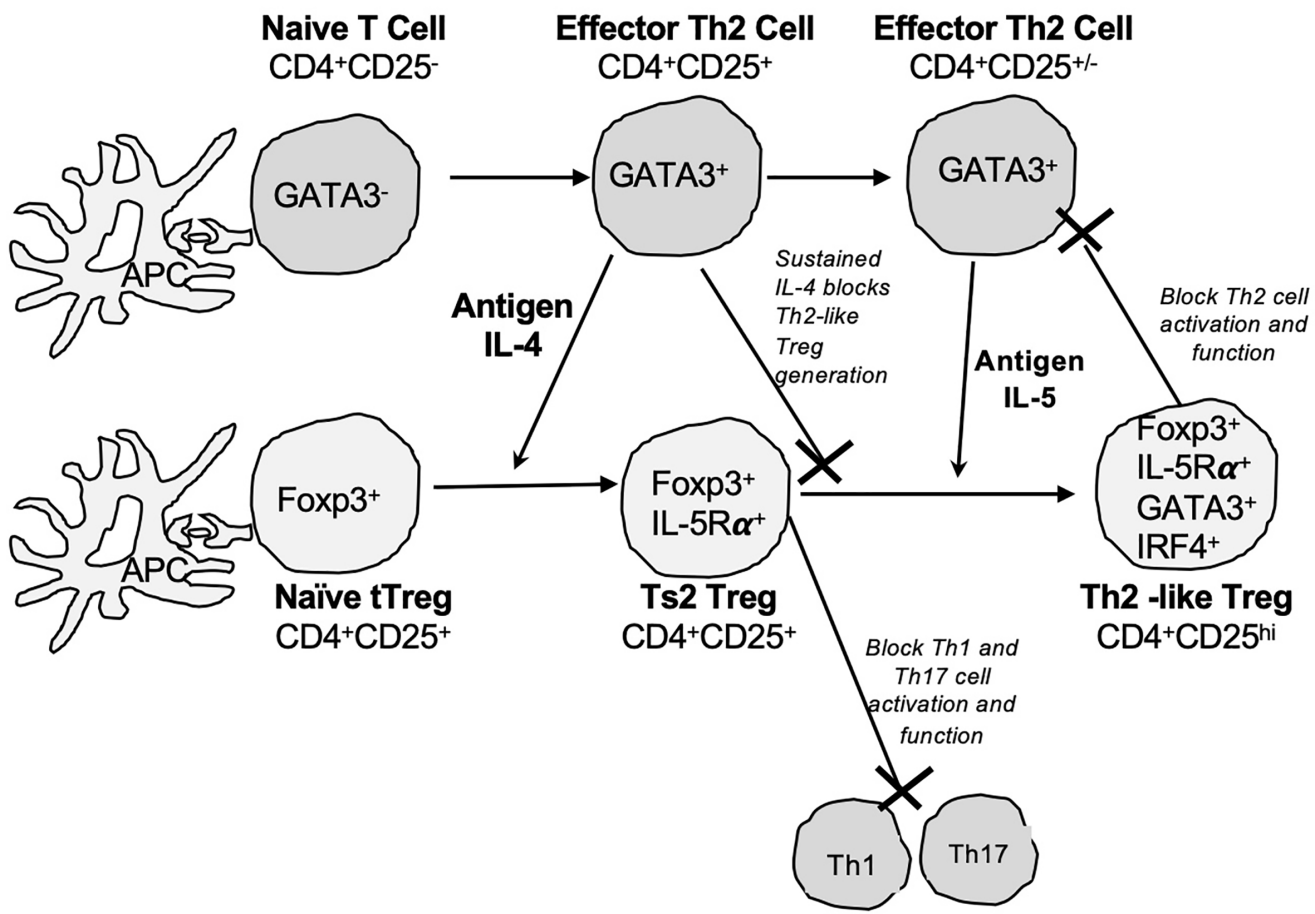
Pathways for activation of naïve CD4^+^CD25^+^Foxp3^+^Treg by Type-2 cytokines and alloantigen. We propose activation of naïve/resting thymic CD4^+^CD25^+^ Treg (tTreg) is driven by cytokines produced by activated effector T cells. The Type-2 cytokine and alloantigen activation pathway of tTreg parallels the activation of effector Th2 cells. Our hypothesis is based on the physiology of immune response. IL-4 is only produced by Th2 cells early in an immune response and late in the response IL-4 is replaced by other Th2 cytokines including IL-5 and IL-13. Our proposed model is that naïve T cells in the rejection response are activated to Th2 cells as well as Th1 cells. Th2 cells express transcription factor GATA-3 and produce Th2 cytokine IL-4 (Top row) in early stages of immune response. This IL-4 activates other naïve T cells to expand the immune response. In parallel, IL-4 also activates naïve/resting CD4^+^CD25^+^Foxp3^+^Treg (tTreg) in a polyclonal fashion and this activation does not require IL-2. tTreg express IL-4Rα and can recognize graft alloantigen in the presence of IL-4 get activated to alloantigen-specific Treg that are induced to express IL-5Rα, the specific receptor for IL-5 (Bottom row). We call these activated antigen-specific CD4^+^CD25^+^Foxp3^+^Treg Ts2 cells (29, 30). Ts2 cells diminish the immune response by inhibition of Th1 and Th17 cells promoting polarization to a Type 2 effector response (30). A sustained Th2 response results in production of IL-5 from Th2 cells (GATA-3^+^), and diminished IL-4 production. In the second step of activation is IL-5 in presence of specific stimulating alloantigen promoting expansion of Ts2 (IL-5Rα^+^Foxp3^+^) to Th2-like Treg. Th2-like Treg express mRNA for *Foxp3, Gata-3*, *Irf4* and *Il5*. Th2-like Treg do not express key markers of Th1-like Treg such as *tbet*, *Ifng. Ifngr or Il12rb2*. The presence of IL-4 during late stage of immune response inhibits induction of Th2-like Treg.

Relevant to this study, we described a second pathway of tTreg activation by Type-2 cytokines (28, 29) (**Figure 1**). Activation of tTreg by IL-4 a Type 2 cytokines is independent of IL-2, as tTreg express the IL4Rα. tTreg cultured with recombinant (r) IL-4 and alloantigen develop into more potent activated Treg that prevent allograft rejection mediated by naïve CD4^+^T cells at a ratio of 1:10 and suppress specific anti-donor responses in MLC at ratios of 1:32 to effector CD4^+^CD25^-^ cells (29). In contrast, fresh naive CD4^+^CD25^+^ cells only fully suppress allograft rejection or anti-donor responses in MLC at 1:1 (26, 31). We called these IL-4 and antigen activated Treg, Ts2 cells (29). Ts2 cells express IL-5Rα, the specific-receptor for the Type 2 cytokine IL-5 (29), and are activated by IL-5 in the presence of specific antigen (30, 32). Using cells from animals tolerant to an allograft, we have shown that IL-5 promotes survival of tolerance-transferring CD4^+^T cells (33) and proliferation of CD4^+^CD25^+^T cells to specific alloantigen (31).

In rats, treatment with rIL-5 reverses autoimmunity (30, 32) and delays neonatal heart graft rejection (34), with inhibition of Th1 and Th17 while sparing of Th2 responses (30, 34). In autoimmunity, the immunosuppressive effect of rIL-5 requires host CD25^+^T cells and IL-4 (30). rIL-5 therapy expands auto-antigen-specific Ts2 cells (30).

In this study we found re-culture of Ts2 cells with specific-antigen and rIL-5, in the absence of rIL-4, induced Th2-like Treg that expressed mRNA for *Gata*-3, Interferon regulatory factor 4 (*Irf*4), II-5Rα and the Th2 cytokine II-5. GATA-3 is the Th2 transcription factor. IRF4 is a transcription factor that is induced by TCR binding to antigen and promotes induction of Th2 cells but not Th1 responses (35). CD4^+^CD25^+^Foxp3^+^ Treg that are activated by Type-2 cytokines (36) depend upon IRF4 to control effector Th2 responses (37).

We hypothesized that rejection responses would activate Th2 cells that produce IL-4 that together with alloantigen, would activate antigen-specific Ts2 cells. Treatment with rIL-5 early post-transplant, in the presence of alloantigeneic stimulation, could promote expansion of these alloantigen specific Ts2 cells and induction of Th2-like Treg. Such Th2-like Treg could complement induction of tolerance by Type-1 cytokine activated tTreg that may occur in parallel as described earlier.

We used Lewis heterotopic heart grafts in F344 hosts (38, 39) where rejection is slow as there is only one class I MHC incompatibility and no class II MHC incompatibilities. We found that treatment with rIL-5 prevented progression of rejection and induced prolonged allograft survival. Monoclonal antibody (mAb) treatment to deplete host CD25^+^ cells or block host IL-4 impaired the rIL-5 effect. Host CD4^+^CD25^+^T cells had specificity for donor antigen when cultured with rIL-5 and expressed molecules associated with Ts2 and Th2-like Treg.

## Materials and Methods

### Animals

F344 (RT1^lvl^) rats were purchased from the Animal Resource Centre (Murdoch, WA, Australia). Lewis (RT-1^l^), PVG (RT1^c^) and DA(RT-1a) rats were bred and maintained in the animal house, Liverpool Hospital. All animals were fed standard chow and given water *ad libitum.* The housing and experiments were in accordance with the Australian Code for the Care and Use of Animals for Scientific Purposes and approved by the Animal Ethics Committee of the UNSW Sydney. Rats that received standard care in the animal house and not given any treatment or alloantigen were considered naïve.

### Heterotopic Heart Graft Procedures

F344 male rats of 200g or more were anesthetized with isoflurane and grafted with heterotopic adult Lewis hearts from 180-230g donors, as described (40). Graft function was monitored daily during the treatment period then two to three times per week. Graft function was scored as *4.* for a strong and fast beat similar to an isograft, *3.* for mild graft swelling and slowing of graft contraction, *2.* for moderate swelling and slowing of graft heartbeat, *1.* for marked swelling and slowing of contraction, *0.5.* for marked bradycardia and minimal and variable contraction, and *0.* if no beat was detected. Total rejection was defined as a score of *0.5* or *0* observed for 10 days. In some tolerance models, graft function can improve days after what appears to be complete rejection. Thus, we observed graft function for weeks after major rejection. Some animals were sacrificed at the end of rIL-5 treatment for histology, as described (17).

### Cytokines

Rat rIL-5 and rat rIL-4 were produced as supernatant from a transfected CHO-K1 cell line that was cultured in serum free medium and activity assessed in bioassays as described (41, 42). Supernatant was concentrated and rIL-5 quantified in a bioassay using the IL-5 dependent cell line B13 (a gift of Dr C. Sanderson, Curtin University, Perth WA, Australia), as described (43–46). 5000 Units of rIL-5 in 0.5 ml was given ipi as a daily dose. 5000 units of rIL-5 per day is well tolerated by rats, induces Ts2 cells to reverse autoimmunity and induces eosinophilia (22).

### Treatment With Monoclonal Antibodies

To deplete CD25^+^cells, the mAb NDS61 (gift of M Dallman, Imperial College London, UK) was given ipi at 7mg/kg daily from day 3 to 17 post-transplantation (30, 47). To block IL-4, 7mg/kg MRCOX81 (gift of N Barclay, Sir William Dunn School of Pathology, Oxford, UK) was given ipi on days 3-8 post-grafting, then every second day until day 15, as described (30, 41). These mAb were produced as described (15).

### Experimental Plan for Transplant Experiments

Five groups of F344 rats with heterotopic Lewis heart grafts (n=4-5) were studied and animals were monitored for heart allograft function. A sham treated group received saline injections daily from Day 7-16 post-transplant and four groups were given rIL-5 daily for 10 days from day 7 to 16 post-transplant. One of these four groups, the short-term rIL-5 treated, had rIL-5 therapy stopped after day 16, this group was repeated three times with results of all animals combined (n=12). Another rIL-5 treatment group, the long term treated group, had rIL-5 therapy continued as three times a week after the day 16. For the remaining two groups that received rIL-5, one was also treated with anti-CD25 mAb and the other with anti-IL-4 mAb, as described above. Some animals were used for histology of the heart graft and/or collection of spleen and lymph node cells for enrichment of CD4^+^CD25^+^T cells for MLC. At the end of experiments, at about 60 days post-transplant, all graft recipients in groups 1, 2 and 3 were sacrificed for FACS, RT-PCR and MLC studies on enriched CD4^+^CD25^+^T cells.

### Histology

Donor heart sections were paraffin fixed and stained with hematoxylin and eosin. The histology images shown in **Figure 5B** were taken by a Leica DFC 450C camera with 20x magnification on a Leica DM 2000 LED microscope as we have described in **Figure 5B** legend. For quantification of areas of myocyte necrosis and mononuclear cell infiltration these paraffin sections were assessed in multiple images taken at 400x magnification on a Zeiss Axioscope A1 microscope (Zeiss, North Ryde, Australia). Image Pro Plus 6.2 software (Media Cybernetics, Rockville, MA) was used to estimate the area of myocytes necrosis and mononuclear cell infiltration, which were expressed as pixels per high power field (HPF).

Immunohistology was performed on 5μM sections of frozen heart allografts cut on a cryostat. Sections were air dried after fixation with acetone for 10min, then stained with a two-step indirect immunoperoxidase technique, as previously described (5, 48). The primary mAb used were W3/25 to CD4, MRCOx8 to CD8 (BD), FJK-16 to Foxp3 and ED1 to CD68 on macrophages (Abcam, Cambridge,cUK), as described (49). The secondary antibodies were HRP labelled anti-mouse Ig (Dako A/s, Glostrup, Denmark). Positive staining was assessed in multiple images taken at 400 X magnification on a Zeiss Axioscope A1 microscope. Image Pro Plus 6.2 software was used to estimate the area of positive staining and was expressed as pixels per high power field (HPF).

### Immunostaining of Lymphocytes

FITC labeled anti-rat mAb used were G4.18 (CD3), W3/25(CD4), MRCOx8 (CD8α), MRCOx39 (CD25, IL-2R alpha chain), MRCOx33 (CD45RA)(BD) and FJK-165 (anti-mouse/rat Foxp3) (eBioscience, San Diego, CA). Staining and analysis of lymphoid cells using a FACScan (BD, San Jose, CA) was as described (19, 50, 51).

### Cell Preparation and Subset Separation

Single cell suspensions from spleen and lymph node were prepared as described (50, 52) and RBCs were lysed with a buffer of 0.83% NH_4_Cl, 0.1%KHCO_3_ and 10mM EDTA at pH 7.2. Cells were re-suspended in PBS/0.4% BSA (MultiGel, Biosciences, Castle Hill, NSW, Australia). Spleen and lymph node cells from three or more animals were pooled to provide sufficient CD4^+^CD25^+^ cells for cultures.

An indirect panning technique was used to deplete CD8^+^T and B cells, as described (14, 50). Briefly, cells were incubated for 30 minutes at 4°C with optimized concentrations of MRCOx8 (an anti-rat CD8α mAb) and MRCOx33 (a rat CD45RA mAb that binds B cells and other cells but not T cells). All mAb were purchased from ThermoFischer. Cells were washed with PBS/0.4%BSA, re-suspended at 2x10^7^ cells/ml and incubated for an hour on Petri dishes (Greiner Bio-one, Kremsmuenster, Austria) coated with both rabbit anti-mouse Ig and rabbit anti-rat Ig (Dako). The unbound CD4^+^ cells were collected and incubated at 4°C for 20 min with PE conjugated MRCOx39 (BD) (an anti-rat CD25 mAb), then washed twice before 8μl/10_6_ cells were incubated for 15 min at 4°C with of anti-PE microbeads (Miltenyi). Enriched CD4^+^CD25^+^ cells were then eluted through a LS MACS column (Miltenyi) and were re-suspended in RPMI 1640 media with 20% Lewis rat serum for culture. Cell subsets were subjected to immunostaining with mAb. Enriched cells were 97-99% CD4^+^ and 80-95% CD25^+^. 60-80% of these CD4^+^CD25^+^T cells were Foxp3^+^.

For RT-PCR and cell culture in MLC, CD4^+^CD25^+^ T cells were re-suspended in PBS/0.4%BSA.

### Assays of Proliferation of CD4^+^CD25^+^T Cells *in MLC*

Stimulator cells were prepared from irradiated (25 gray) thymus cells, as described^19.^ In each experiment parallel cultures with self (F344), specific donor (Lewis), third party (PVG) stimulator cells or no stimulator cells were performed. Cell culture medium was RPMI 1640 (GIBCO, Grand Island, NY) supplemented with 100 ng/ml penicillin, 100 U/ml streptomycin (Glaxo, Boronia, Victoria, Australia), 2 mM L-glutamine, 5x10^-5^M 2-mercaptoethanol (Sigma), and 20% Lewis rat serum. 20% Lewis rat serum produces low background stimulation in autologous controls (19). Cultures with 5-6 replicates for each experimental group were set up in U-bottom micro-titer plates (Linbro, Flow Labs, VA) containing 2 x 10^4^ stimulators cells and 1 x 10^5^ CD4^+^CD25^+^ cells/well in a total volume of 200 μl. To assess the effects of rIL-5 on proliferation of these cells, 200 U/ml of rIL-5 was added to some cultures, as described (30, 32). Where stated CD4^+^CD25^+^T cells from naïve animals were cultured with rIL-4 (200 units/ml) as described (29).

Cells were cultured at 37°C in humidified air containing 5% CO_2_ for 4 days, the peak of CD4^+^CD25^+^T cell proliferation (26). 0.5µCi ^3^H-thymidine (TRK-120, Amersham, Arlington Heights, IL) was added 16hr prior to harvesting with a Tomtec Cell Harvester (Flow Lab, Ayrshire, Scotland). Proliferation was assayed by adding liquid scintillation fluid before counting on a beta counter (1450 Microbeta Plus, Beckman Instruments, Palo Alto, CA). Each experiment has 5-6 replicates and results were expressed as cpm and presented as mean +/- standard deviation (SD). Counts of <400/min were considered within the range of background

The effect of rIL-5 on CD4^+^CD25^+^T cells proliferation in culture was calculated as a Stimulation Index using the formula: proliferation of cells with rIL-5 to a defined antigen divided by proliferation to the same defined antigen without rIL-5.

### RT-PCR of Cytokines and Cytokine Receptors

mRNA extraction from cells and reverse transcription to DNA were as described (21). Primers for rat *Foxp3, Gata3, Tbet, Il2, Il4, Il5, Ifng, Ifngr, Il5ra, Il12rb2 and Gapdh* were as previously reported (28–30, 53). The primers for *Irf4* were F-TGTCCTCCGTGAGCTGTCT; R- CCTGGATCGGCTCCTCTATG, as described (49). **The panel of molecules examined were selected for their relevance to Treg activation**. Real-time PCR was performed as described (54) with a Rotorgene (Corbett Research) and SYBR Green I detection. Sensimix Taq polymerase (BioLine) was used according to manufacturer’s instructions. Copy numbers of each gene was derived from a known standard curve performed in parallel and normalized against *Gapdh* expression.

### Statistics

Parametric data were expressed as mean ± standard deviation. Results from repeat experiments were pooled, with replicates of ≥ 3 in each experiment. Means were compared using t test with GraphPad Prism (Graphpad Software Inc, La Jolla, CA). Statistical significance was set at p< 0.05.

## Results

### RT-PCR of mRNA From Naïve *CD4^+^CD25^+^* Cells After Culture in MLC With Alloantigen and Type 2 Cytokines

**Figure 2:**
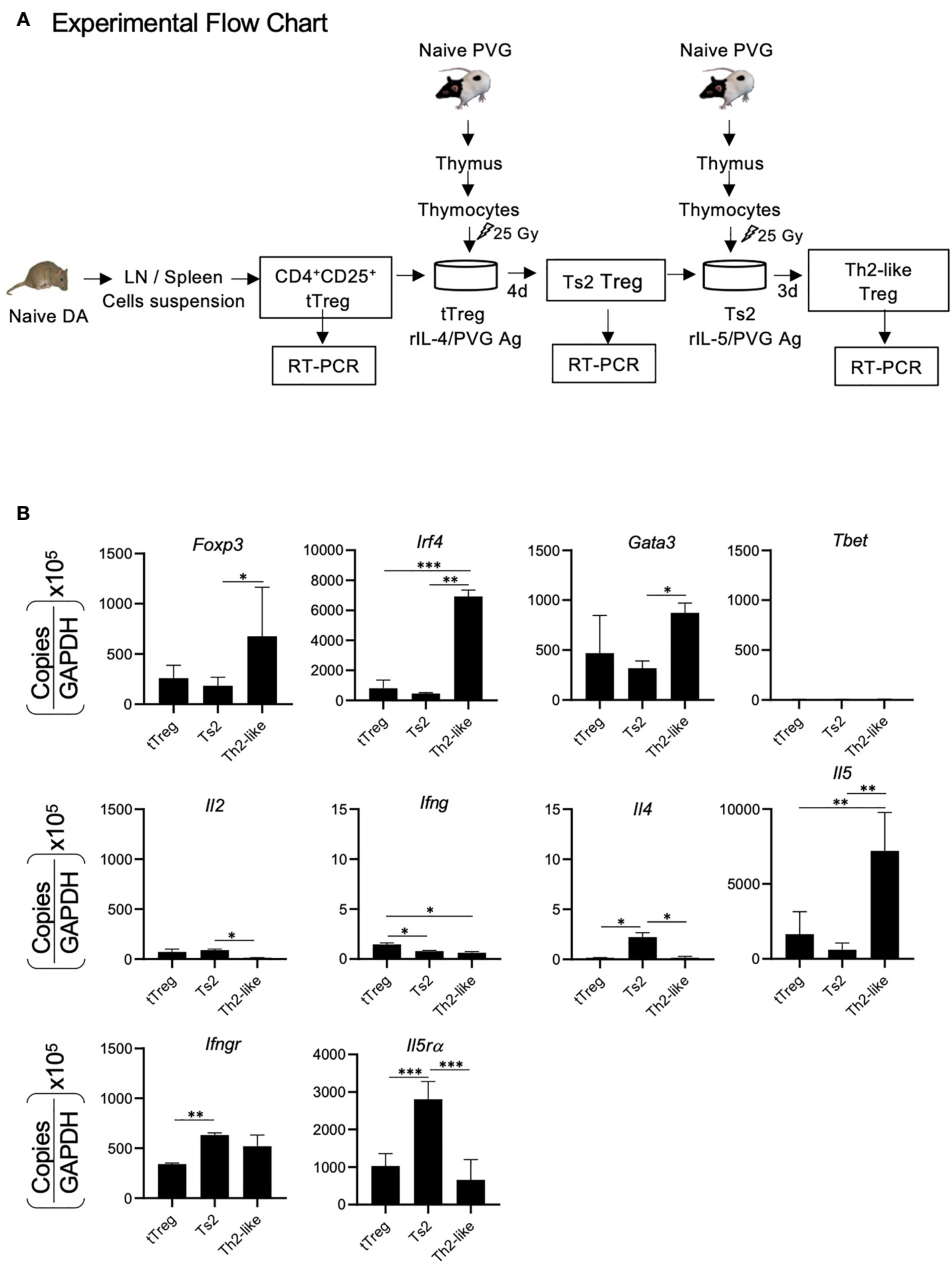
RT-PCR of mRNA from CD4^+^CD25^+^T cells demonstrating changes in naïve CD4^+^CD25^+^T cells cultured with alloantigen and Type-2 cytokines. **(A)** Experimental Flow Chart. CD4^+^CD25^+^T cells from naïve DA rats were enriched and cultured with fully allogeneic PVG thymic stimulator cells and rIL-4 for 4 days to induce Ts2 cells that express II-5Rα. These Ts2 cells were re-cultured for 3 days with same alloantigen and rIL-5 to induce the Th2-like Treg. RT-PCR was performed on mRNA from fresh naïve CD4^+^CD25^+^Treg and cultured activated Ts2 and Th2-like Treg to examine for transcriptions factors *Foxp3, T-bet, Gata-3, Irf4;* cytokines *Il2, Il4, Il5, Ifng* and cytokine receptors *Il5ra, Ifngr*. **(B)** Results of RT-PCR of tTreg, Ts2 and Th2 like Treg. Data shown is a combination of results from three separate experiments. Data expressed as copies for relevant molecule divided by copies of Gapdh, multiplied by 10^5.^ *p < 0.05, **p < 0.01, ***p < 0.001. The Th2-like Treg had greater expression of *Foxp3, Irf4* and *Il5* than fresh naïve CD4^+^CD25^+^T cells and Ts2 cells. They had more *Gata-3* than Ts2 cells*. Il5ra* was induced in Ts2 cells but wa*s* not sustained in Th2-like Treg*. T-bet, Ifng* and *ll4* were low in all samples and there was minimal *Il2* (<100 copies). Expression of *Irf4* and *Il5* were used as markers of Th2 like Treg. The changes associated with Th1-like Treg have been described (28).

To establish the changes in alloantigen activated Treg during Type 2- responses, we examined cytokines, cytokine receptors and transcription factors that are induced after naïve/resting CD4^+^CD25^+^T cells are cultured first with alloantigen and rIL-4 and later in a second culture with specific alloantigen and rIL-5. The experimental protocol is illustrated in **Figure 2A**. The hypothesis was that rIL-4 and alloantigen would activate naïve/resting CD4^+^CD25^+^Treg to Ts2 cells expressing IL-5Rα that would proliferate when stimulated by specific-alloantigen and rIL-5 (29, 30, 32) and develop into Th2-like Treg.

CD4^+^CD25^+^cells from naïve DA rats were cultured for 4 days with fully allogeneic PVG stimulator cells and 200 units of rIL-4 as described (29) to induce Ts2 cells. These Ts2 cells were washed and re-cultured with 200 units of rIL-5 and the same alloantigen to induce Th2-like Treg. Combined results from three separate experiments of RT-PCR of mRNA of these cells are shown in **Figure 2B**. The Th2-like Treg had increased expression of mRNA for the transcription factors *Foxp3, Irf4*, and *Gata-3*, but had no induction of *tbet. il5* but not *il4, il2* or *ifng* was induced in these cells. Compared to starting CD4^+^CD25^+^ cells from naïve rats where naïve/resting tTreg (CD4^+^CD25^+^Foxp3^+^Treg) forms a major part, *Il5ra* expression was increased in Ts2 cells, but this increase was not sustained in Th2-like Treg. We used expression of *Irf4* and *Il5* as markers of Th2-like Treg induction.

### Effect of rIL-5 Treatment on Lewis Heart Graft Rejection in F344 Rats

**Figure 3:**
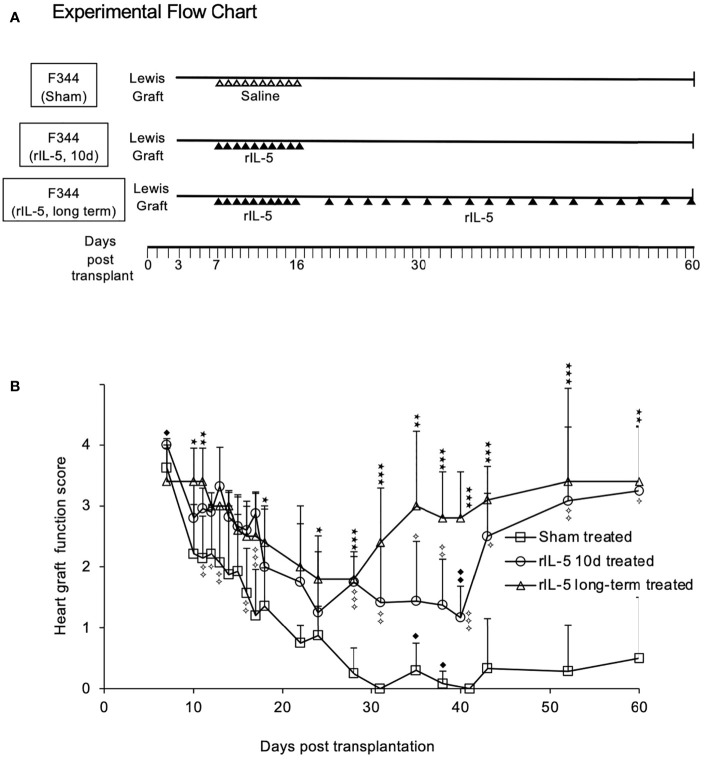
IL-5 treatment prevents rejection of Lewis heart grafts in F344 hosts. **(A)** Experimental Flow Chart. Groups of F344 rats with heterotopic Lewis heart grafts were studied. Sham treated rats received saline (ipi) daily from day 7-16 post-transplantation. One treatment group (short-term rIL-5) received 5000 units/day of rIL-5 (ipi) from day 7 to 16 post-transplant. The second treatment group (long term IL-5) received rIL-5 therapy 5000 units/day from day 7-16 and beyond 16 days had three ipi of 5000 units rIL-5 per week until 60 days post transplantation. **(B)** Monitoring of F344 rats with Lewis heterotopic cardiac allografts. Severity of rejection was assessed by palpation and scored on a semi-quantitative scale, described in methods. Severity of rejection scores in each group was expressed as mean ± standard deviation. Sham treated (□) received daily injections of 0.5ml of normal saline ipi starting at day 7 through to day 16 (n=5). All grafts had severe rejection by day 21. After 28 days, the mean score was <0.5 and there was no recovery of graft function. Short term rIL-5 treatment for 10d (○). 5000 units of rat rIL-5 was given daily ipi in 0.5ml from day 7 through to day 16 (n= 12), as described (22). Rejection was significantly less than sham treated on day 16 (p<0.01) and at day 17 (p<0.001). From day 28, graft function improved and was significantly greater than sham treated controls until day 60 (p<0.05). Significance compared to sham treated controls; ✧ p<0.05, ✧✧ P<0.01; ✧✧✧ p<0.001. Long-term rIL-5 treated (△). 5000 units of rat rIL-5 in 0.5ml was given ipi in 0.5ml daily from day 7 through to day 16, then 3 times a week until day 60 (n= 5). Rejection in this group was significantly less (p<0.01) than sham treated group from days 19 until day 63. Rejection was significantly less than the group treated with rIL-5 for 10 days from day 32 until day 50 (p<0.05). The long-term rIL-5 treated group had less rejection than the short-term rIL-5 treated group at day 34 and day 38 (p<0.05) and at day 40 (p<0.01). By day 43, both rIL-5 treated groups had a mean graft score of *3*. Significance compared to sham treated; *p < 0.05, **p < 0.01; ***p < 0.001. Significance compared to 10 day rIL-5 treated ◆ p<0.05, ◆◆ p<0.01.

**Figure 4:**
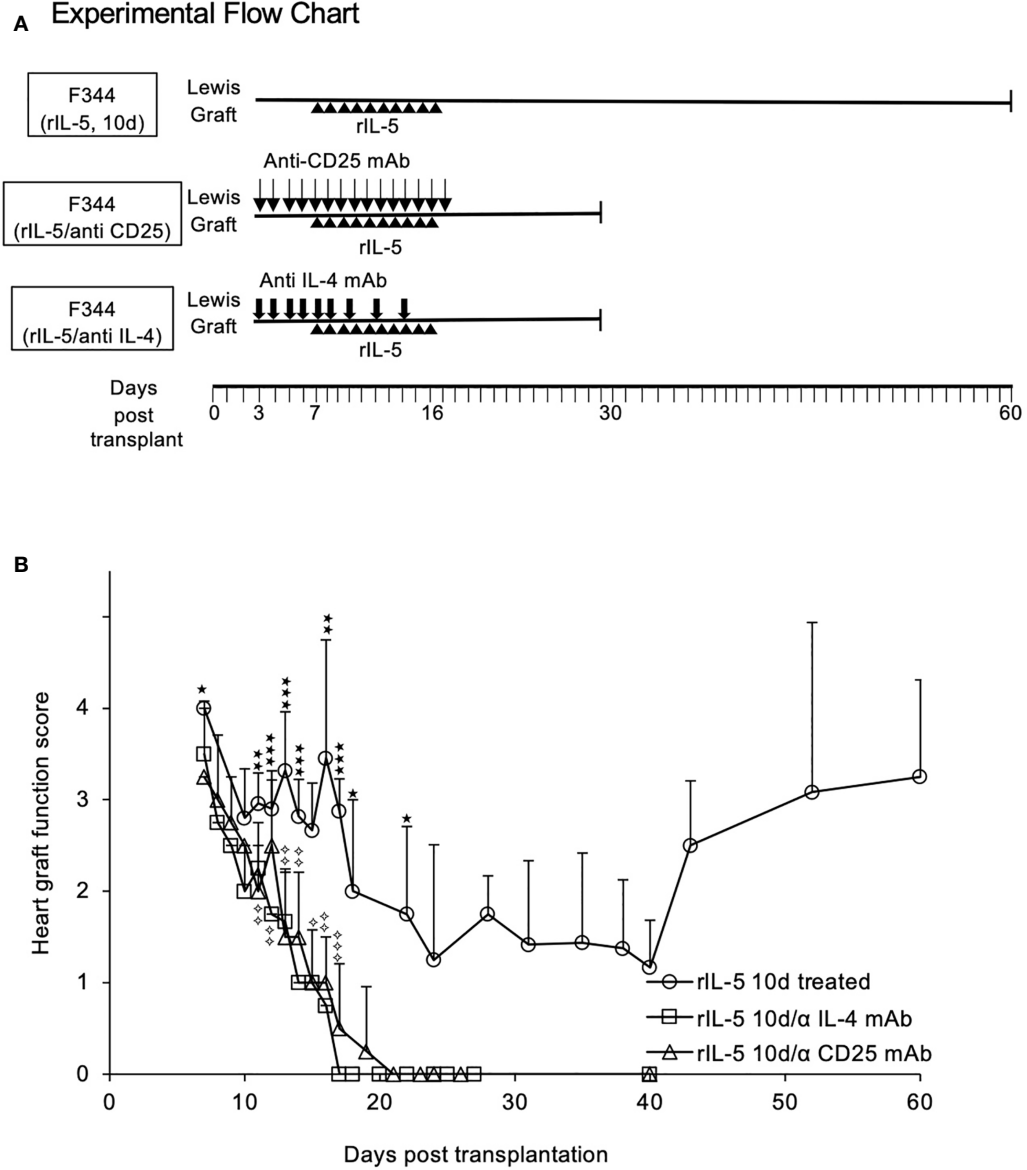
Blocking IL-4 or depleting CD25^+^ cells prevents rIL-5 treatment inhibiting rejection of Lewis heterotopic heart allografts in F344 recipients. **(A)** Experimental Flow Chart. All groups received 5000 units of rat rIL-5 daily (ipi) in 0.5ml. One group was treated with anti-CD25 mAb (NDS61) (n=4) and another with MRC OX81 (n=4), as described in methods. A control group received rIL-5 treatment from day 7-16 post transplantation, as in **Figure 3**, with no blocking mAb (○). Rejection scores are expressed as mean ± standard deviation. **(B)** Graft survival anti-CD25 mAb treated. Anti-CD25 treated (△) rats had more severe graft rejection than rIL-5 treated alone with significant differences from day 15 onwards to day 40 post-transplant (p<0.05). All grafts had fully rejected by day 19, and none recovered. Significance compared to rIL-5 treated controls; ✧ p<0.05, ✧✧ p<0.01; ✧✧✧ p < 0.001. Graft Survival anti-IL-4 treated. Animals treated with anti-IL-4 mAb (□) rejected their transplants more rapidly than rats treated with rIL-5 alone, with significant differences from day 9 (p<0.05) and on all subsequent days (p<0.01). All grafts were fully rejected by day 17 and none recovered. Significance compared to rIL-5 treated controls; *p < 0.05, **p < 0.01, ***p < 0.001. Taken together, these studies showed host CD25^+^T cells, presumably naïve Treg, were required as was host IL-4 to induce a state where rIL-5 therapy could delay rejection and promote long-term allograft survival.

Our hypothesis is that during a rejection response, some Th2 cells will be activated to produce IL-4 that with donor antigen would activate naïve/resting CD4^+^CD25^+^Foxp3^+^Treg to Ts2 Treg as proposed in **Figure 1**. Lewis heterotopic cardiac allografts into F344 rats are slow to reject as there is only a single class I MHC and multiple minor incompatibilities with no class II MHC incompatibility (27, 28). The model is delayed acute rejection with T cell activation and infiltration. The experimental plan is shown in **Figures 3A** and **4A**. Graft function was scored using a semi-quantitative scale described in methods and mean heart graft function score are presented on y-axis (**Figures 3B**, **4B**).

Rejection in sham treated hosts (n=8) caused a decline in graft function after day 10, with complete rejection by day 31 (**Figure 3B**). No rats in this sham treated control group recovered to have significant function, with graft function scores of 0.5 or 0.

Short-term rIL-5 treatment (n=12) was 5000 units ipi daily for 10 days between 7 and 16 days post-transplantation (**Figure 3A**). In the long-term treatment group (n=5), rIL-5 therapy was continued (ipi) three times a week immediately following the daily rIL-5 from 7 to 16 days post-transplant (**Figure 3A**). In both rIL-5 treated groups, graft function scores were higher than in sham treated rats at all time points beyond day 10 post-transplant (p<0.01)(**Figure 3B**). rIL-5 treatment preserved graft function, with all grafts scoring ≥2 until cessation of rIL-5 treatment at day 16. The graft function score was significantly higher than sham treated group, p<0.01 at day 16 and p<0.001 at day 17 (**Figure 3B**).

Heart graft function in both rIL-5 treatment groups stabilized around 20 days post-transplant then improved. The group that received long-term treatment with rIL-5 therapy had more rapid improvement in graft function, with scores significantly higher than sham treated controls at all time points beyond day 22 (p<0.05). Compared to 10-day treatment group, the long-term rIL-5 treated group had higher graft function scores on day 35 (p=0.05) and 40 (p<0.01) (**Figure 3B**). By day 43, both rIL-5 treated groups had a mean graft function score of 3. At the end of monitoring on day 60, 3 of 5 long-term rIL-5 treated rats had an excellent graft function score of *4* and another rat had a score of *3* (**Figure 3B**). This level of heart graft function is consistent with operational transplant tolerance and similar to long-term syngeneic heart graft function in this rat allograft model, as observed in previous studies.

### Depletion of CD25^+^ Cells Prevented rIL-5 Treatment Slowing Rejection

The rationale for rIL-5 treatment was to expand CD4^+^CD25^+^Foxp3^+^Treg that had been activated by IL-4 produced in the early rejection response. NDS61, a mAb to rat CD25, depletes CD4^+^CD25^+^T cells in rats (47) and prevents rIL-5 treatment inhibiting autoimmune responses (30, 32). To demonstrate a role for CD25^+^ cells, we depleted these cells by ipi of NDS61 daily from 3 to 17 days post-transplantation, as illustrated in **Figure 4A**. Hosts depleted of CD25^+^T cells and treated with rIL-5 rejected their allografts faster, with all allografts fully rejected at day 19 (n=4) (**Figure 4B**). No graft function was detected in any animal treated with NDS61 and rIL-5 after day 20 and there was no recovery in graft function in the next 10 days. Graft rejection was more severe in anti-CD25mAb and rIL-5 treated rats (**Figure 4B**) than in sham treated from day 14 to 19 (p<0.05) (**Figure 3B**). This suggests CD25^+^cells are activated during rejection and slow the progress of rejection.

### Blocking IL-4 Inhibited the Effects of rIL-5 Treatment on Preventing Rejection

MRCOx81, a mAb that blocks IL-4 (40, 41), was administered daily from day 3-8 then on days 10,12,14 post-transplantation, as illustrated in **Figure 4A**. Anti-IL-4 mAb treatment also led to accelerated rejection and abolished the effect of rIL-5 treatment on allograft survival (n=4) (**Figure 4B**). All rats totally rejected their heart grafts by day 17 and there was no recovery in graft function over the next 10 days. All MRCOx81 treated rats rejected faster than sham treated controls (**Figure 3B** and **Figure 4B**). MRCOx81 and rIL-5 treated group had significantly lower graft function scores (**Figure 4B**) compared to those from rats treated with rIL-5 alone, on all monitoring days from day 11 (p<0.01)(**Figure 4B**).

### Histology of Heart Grafts in rIL-5 Treated Hosts: rIL-5 Treatment Reduced Mononuclear Cell Infiltration and Damage to Myocytes

**Figure 5:**
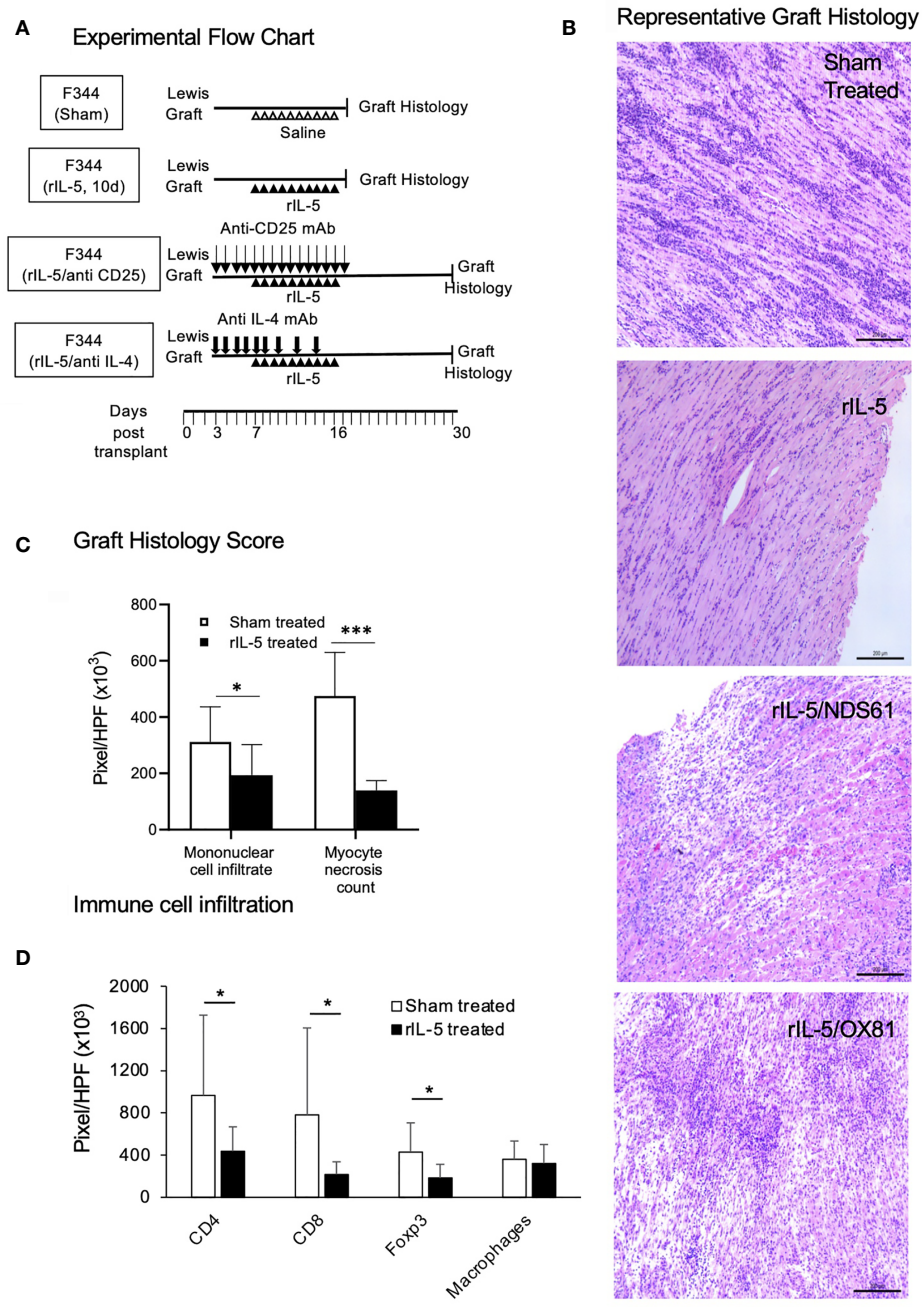
rIL-5 treatment reduces myocyte necrosis and mononuclear cell infiltrates in Lewis heart grafts transplanted to F344 hosts. **(A)** Experimental Flow Chart. Representative animals from sham treatment and 10 day rIL-5 treatment (**Figure 3**) groups were sacrificed for study of histology, myocyte damage, and mononuclear cell infiltrates in their cardiac allogafts. Some animals from anti-CD25 and anti-IL-4 mAb therapy together with rIL-5 treatment groups (**Figure 4**) were sacrificed for histological studies. **(B)** Photomicrographs of H&E sections of Lewis cardiac allografts from F344 hosts, Samples were taken at days 17-19 post-transplant shortly after daily rIL-5 treatment had stopped. Images taken by a Leica DFC 450C camera with 400X magnification on a Leica DM 2000 LED microscope. Heart grafts from sham-treated hosts had large areas of mononuclear cell infiltration and scattered infiltrate between myocytes (Top panel). There were wide areas of myocyte necrosis. Heart grafts from animals treated with rIL-5 had minimal mononuclear infiltration between myocytes and minimal myocyte necrosis (second panel). Grafts from hosts treated with rIL-5 that also received NDS61 an anti-CD25 mAb to deplete Treg (third panel) and MRCOX81 mAb to block IL-4 (bottom panel) had large areas of myocyte necrosis and cell infiltration. **(C)** Areas of mononuclear cell infiltrate and myocyte necrosis was assessed as pixels per high power field (HPF) in multiple images taken at 400 X magnification on a Zeiss Axioscope A1 microscope (Zeiss, North Ryde, Australia) using Image Pro Plus 6.2 software (Media Cybernetics, Rockville, MA). Data expressed as mean ± SD, *p < 0.05, ***p < 0.001. Area of mononuclear cell infiltration measured in pixels was significantly less in grafts in rIL-5 treated rats than those from sham treated rats; 193,883 ± 108,701 *vs* 311,4112 ± 124,968 (p = 0.03) (**Figure 3C**). Area of myocytes necrosis measured in pixels was significantly lower (p<0.00011) in heart grafts from rIL-5 treated rats than in hearts from sham treated controls (**Figure 3C**). **(D)** Immunostaining for mononuclear cells in heart grafts from F344 rats showing comparison of cell infiltrate in grafts from rIL-5 treated animals to those from sham treated. Grafts from rIL-5 treated animals had significantly reduced area of CD4^+^ cells (p<0.05), CD8^+^ cells (p<0.05), and Foxp3^+^ cells (p<0.05) compared to those from sham treated animals. The ratio of Foxp3^+^ cells in CD4^+^ cells was similar in rIL-5 treated and sham-treated rejection controls. There was no difference in infiltration of ED1^+^ macrophages. Photomicrographs of these stained sections are in **Supplementary Figure 2**.

The experimental protocol for obtaining Lewis heart graft tissue from F344 rats for histology is illustrated in **Figure 5A**. Heart grafts from rIL-5 treated rats taken at day 16 post-transplant had good cardiac myocyte preservation with scattered mononuclear cells infiltration (**Figure 5B**). In contrast, grafts from sham treated rats had wide-spread myocyte necrosis and large infiltrates of mononuclear cells. Additional examples are in **Supplementary Figure 1**. Donor Lewis hearts in F344 recipients treated with rIL-5 and either anti-CD25 or anti-IL-4 mAb had massive areas of myocyte necrosis with dense infiltrates of mononuclear cells, however these heart grafts were taken at the end of the experiment at day 30 not at day 17 post-transplant (**Figure 5B**).

Image analysis of donor hearts showed the pixels occupied by necrotic myocytes was less in rIL-5 treated 139,475 ± 35,078 than in sham treated controls 474,969 ± 154,423 (p=0.00011); anti-IL-4 mAb and rIL-5 treated 436,217 ± 138,148 and anti-CD25 mAb plus rIL-5 treated 536,889 ± 272,577 (**Figure 5C**). The area of mononuclear cells measured by pixels was less in rIL-5 treated 193,883 ± 108,701 than in sham treated controls, 311,4112 ± 124,968 (p=0.03), anti-IL-4 mAb and rIL-5 treated 269,521 ± 35,636 and anti-CD25 mAb plus rIL-5 treated 249,281 ± 102,820 (**Figure 5C**). The mAb treated animals grafts were collected two weeks longer post-transplant and had established rejection, thus this data is not directly comparable.

Characterization of the mononuclear cell infiltrate in heart grafts using immunostaining with mAb, (**Figure 5D**), showed that compared to grafts from sham treated hosts that were rejected, rIL-5 treated grafts had significantly fewer CD8^+^ cells (p=0.02), CD4^+^ cells (p<0.05) and Foxp3^+^ cells (p= 0.05). 44% of CD4^+^ cells in IL-5 treated expressed Foxp3, whereas 41.9% expressed Foxp3 in rejected grafts. Representative sections are shown in **Supplementary Figure 2**. There was no difference in the infiltrate of ED1^+^ macrophages between sham treated and rIL-5 treated.

Thus, rIL-5 treatment preserved the heart graft from injury and markedly reduced the mononuclear cell infiltrate compared to grafts from sham-treated rats. The benefits of rIL-5 treatment were abolished by treatment with anti-CD25 mAb or anti-IL-4 mAb (**Figures 5A–D**).

### Effect of Treatment With rIL-5 for 10 Days on CD4^+^CD25^+^T Cells in Peripheral Lymphoid Organs of Hosts

**Figure 6:**
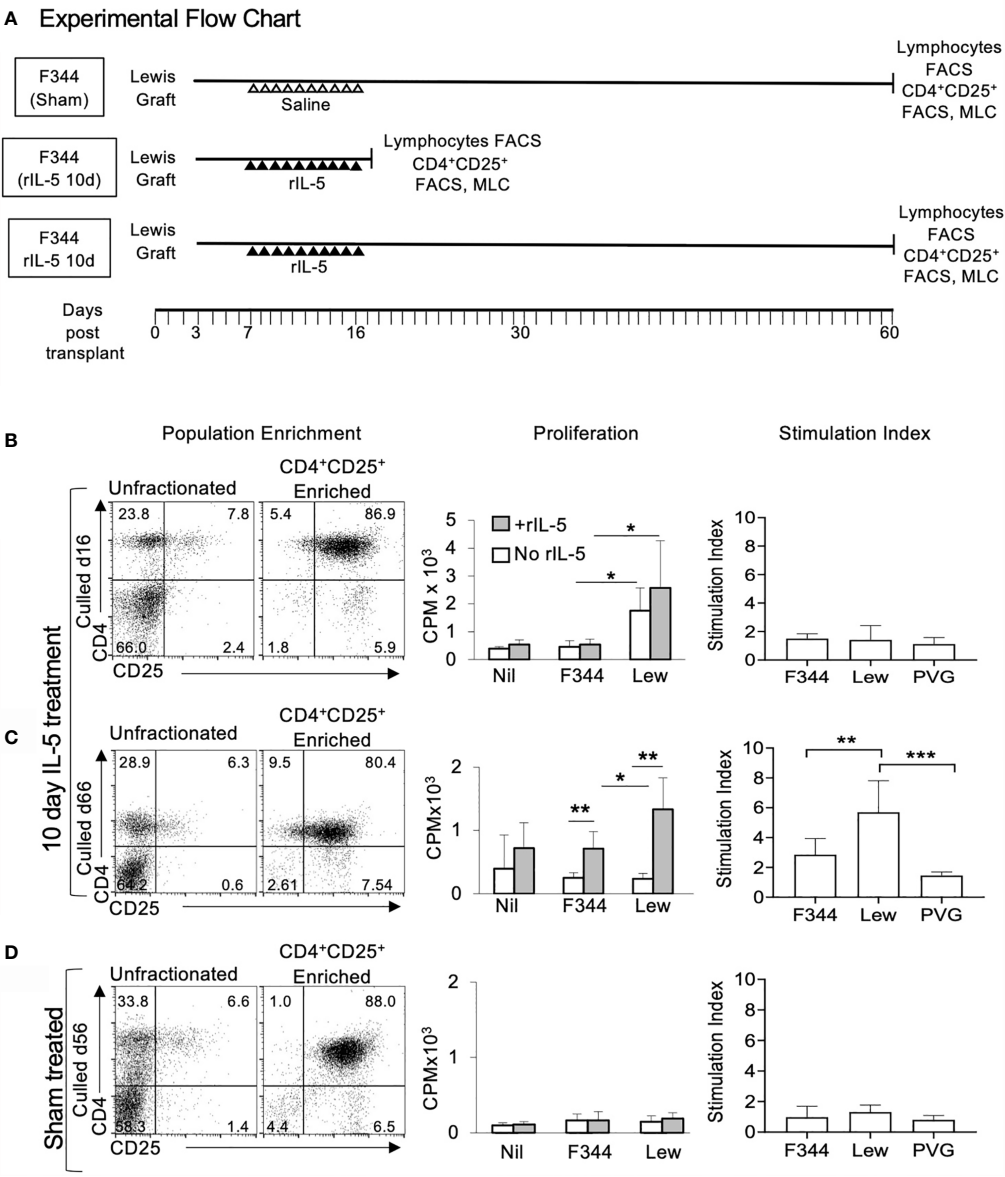
Flow cytometry profiles and proliferation of CD4^+^CD25^+^ T cells from spleen and lymph nodes from F344 recipients of Lewis cardiac allografts. **(A)** Experimental flow chart with animal treatment and collection time of peripheral lymphoid tissues for FACS and MLC. All cell donors were F344 grafted with a Lewis heterotopic heart transplant. Cells from rIL-5 treated were taken at 16 days post transplantation at the end of 10 days rIL-5 treatment, or at 66 days post-transplant. Sham treated recipients’ cells were taken at 56 days post-transplant. **(B–D)** Enriched CD4^+^CD25^+^T cells (left column) from lymph nodes and spleens from Lewis allograft bearing F344 recipients were examined for their capacity to proliferate in MLC in response to no stimulator cells (Nil), or stimulator cells from self (F344), specific donor (Lewis) or third party PVG. The understanding of the current findings is dependent upon our previous findings. First, naïve CD4^+^CD25^+^T cells, in the absence of rIL-2 or IL-4 have a very small response to alloantigen, and none to self. CD4^+^CD25^+^T cells from tolerant hosts do not respond to the tolerated donor strain but they do respond to third party. The proliferation of CD4^+^CD25^+^T cells from tolerant hosts to specific donor, but not to self or third party is enhanced by addition of cytokines such as rIL-5. Effect of rIL-5 on proliferation of CD4^+^CD25^+^T cells to self and specific donor is shown in middle column. Proliferation to third party PVG, which is fully allogeneic, is much larger to self and Lewis (data not shown). Stimulation indices were calculated as proliferation with rIL-5 in culture divided by the proliferation to the same donor stimulator cells with no rIL-5 (n=-6). **(B)** Cells from hosts treated with rIL-5 taken at day 16 post-transplant. CD4^+^CD25^+^T cells represented 7.8% of unfractionated lymphocytes and 87% of enriched cells (left panel). The enriched CD4^+^CD25^+^ T cells proliferated to specific donor, but not to self (middle panel). This proliferation was slightly enhanced by adding rIL-5 to cultures, but not significantly when assessed as Stimulation Index (right panel). **(C)** Cells from hosts treated with rIL-5 for 10 days and culled 66 days post-transplant, had 6.3% CD4^+^CD25^+^ cells (left panel). CD4^+^CD25^+^T cells did not respond to self or specific donor (middle panel). The proliferation to specific donor was enhanced significantly by adding rIL-5 to the culture (middle panel) as illustrated by Stimulation Index (right panel). The response to self or third-party was not enhanced by rIL-5 (right panel). This is consistent with our hypothesis that alloantigen specific CD4^+^CD25^+^T cells become dependent upon IL-5 for expansion. **(D)** Cells from hosts given sham treatment taken at 56 days post-transplantation had similar proportions of CD4^+^CD25^+^ cells (6.6%) to animals treated with rIL-5. However, these cells did not respond to specific donor alone, and rIL-5 did not enhance proliferation (middle and right panel), indicating absence of alloantigen-specific Treg that depend upon IL-5. *p < 0.5, **p < 0.01, ***p < 0.05.

**Figure 7:**
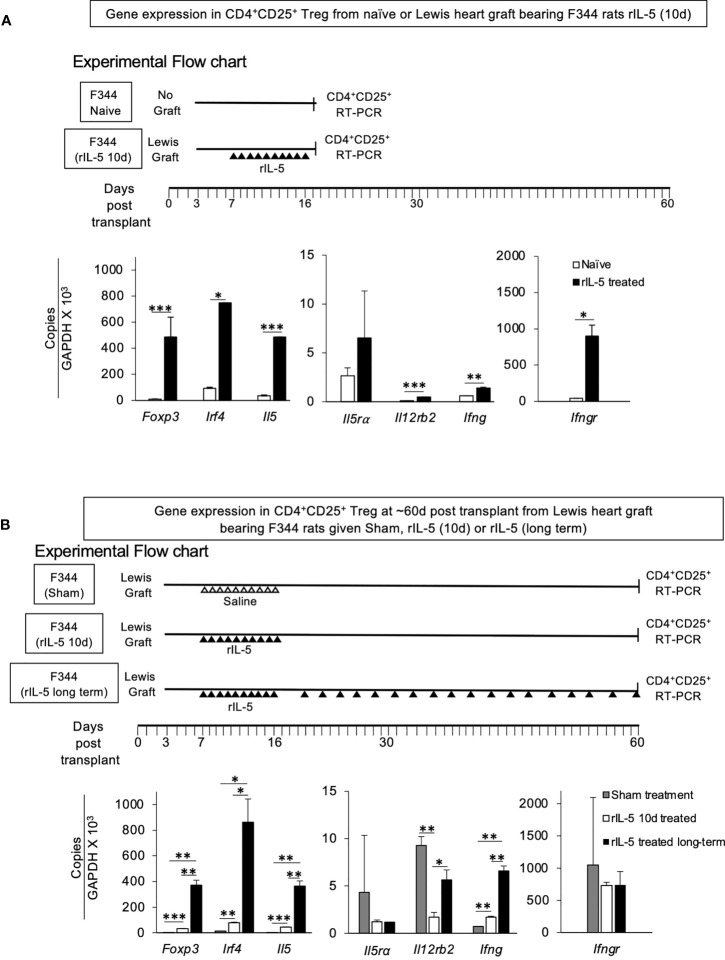
RT-PCR assays of mRNA for transcription factors, cytokines and cytokine receptors in CD4^+^CD25^+^T cells from F344 rats. **(A)** Comparison of RT-PCR of mRNA of CD4^+^CD25^+^T cells from naïve F344 to those from F344 rats bearing Lewis heart graft treated with rIL-5 for 10 days. CD4^+^CD25^+^ cells were enriched from lymph node and spleens of F344 rats as described in methods and subjected to mRNA extraction. mRNA prepared at 16 days post transplantation from F344 rats bearing Lewis heart grafts and treated with rIL-5 for 10 days was compared to that from naive F344 rats that had no transplant and no treatment (Experimental Flow Chart). mRNA was subjected to cDNA extraction followed by RT-PCR of transcription factors, cytokines and cytokine receptors associated with activation of naive CD4^+^CD25^+^ T cells by alloantigen and either IL-2 (*Ifngr, Il12rb2)* or IL-4 (*Irf4* and *Il5ra*). CD4^+^CD25^+^T cells from rIL-5 treated graft bearing hosts had more *Foxp3* consistent with activation of Treg, and more *Il5*, and *Irf4*, consistent with induction of Th2-like Treg. There was also induction of *Ifngr, Ifng* and *Il12rb2* consistent with induction of Treg by Type-1 cytokines (28, 29). *p < 0.05, **p < 0.01, ***p < 0.001. **(B)** Comparison of RT-PCR of mRNA from CD4^+^CD25^+^ cells from F344 hosts with Lewis heart grafts at >56 days. CD4^+^CD25^+^T cells from spleen and lymph nodes of F344 rats treated with rIL-5 for short term (10 days) or long-term were compared to those from sham treated hosts (Experimental Flow Chart). CD4^+^CD25^+^T cells in long-term rIL-5 treated animals were not increased and was ~6.4% (data not shown), similar to those from short-term rIL-5 treated and sham- treated rats (**Figure 6**). CD4^+^CD25^+^T cells from long-term treatment group had greater expression of *Foxp3, Irf4* and *Il5* than cells from recipients where rIL-5 treatment was given only for 10 days that stopped at 16 days post transplantation. Cells from sham treated rats had less expression of Th2-like Treg markers than those from rats given short term rIL-5 treatment. Thus, continued rIL-5 treatment strongly retained the Th2-like Treg phenotype. *p < 0.05, **p < 0.01, ***p < 0.001.

The source of lymphocytes for these studies is illustrated in **Figure 6A**. We examined CD4^+^CD25^+^ cells from graft bearing hosts to examine if IL-5 administration resulted in *in vivo* activation of Ts2 cells and/or Th2-like Treg as assessed by *in vitro* proliferation with rIL-5 (**Figures 6B–D**) and RT-PCR of key markers (**Figures 7A, B**).

Spleen and lymph node cells from rats treated with rIL-5 for 10 days were examined either at the end of rIL-5 treatment on day 16 (**Figure 6B**), or on day 66 (**Figure 6C**) post-transplantation. Cells from sham treated F344 rats with Lewis heart graft were assessed at 56 days post-transplantation (**Figure 6D**). The proportion of CD4^+^CD25^+^cells in rIL-5 treated rats was 6.3-7.8% (**Figures 6B, C**) compared to 4% in sham-treated controls (**Figure 6D**). Foxp3^+^ cells were 2.8% - 4% in rIL-5 treated rats and 6.6% in sham-treated controls (data not shown).

The enriched CD4^+^CD25^+^ cells in all three groups were 80-88% CD4^+^CD25^+^cells (**Figures 6B–D** left panel), 94-99% CD3^+^, <2.4% CD8^+^, and 61-70% Foxp3^+^ cells (data not shown). This is within the standard enrichment of murine Treg using CD25. Thus, 30-40% of cells in the enriched CD4^+^CD25^+^T cells were Foxp3^-^ and not necessarily T regulatory cells. They may include activated effector CD4^+^T cells.

Enriched CD4^+^CD25^+^ cells were tested for proliferation in MLC (**Figures 6B–D**, middle and right panel) and their mRNA tested by RT-PCR (**Figures 7A, B**). Enriched CD4^+^CD25^+^T cells from rIL-5 treated hosts taken 16 days post-transplant (**Figure 6B**, middle panel), in absence of rIL-5 in culture, had a greater response to Lewis, than to self (F344) or third party. Such proliferation to graft alloantigen suggested increased numbers of cells activated by graft alloantigens. rIL-5 in cultures partially enhanced responses to specific donor Lewis, but this was not significant, as seen in the Stimulation Index (**Figure 6B**, right panel). rIL-5 in culture also did not enhance the response to self (F344) or third party (PVG) (**Figure 6B**, middle and right panel).

Enriched CD4^+^CD25^+^ cells (>88%) from F344 rats with Lewis heart grafts, treated for 10 days with rIL-5, at day 66 post-transplant had no proliferation to specific-donor in the absence of rIL-5 (**Figure 6C**, middle panel). This was consistent with our previous observations that Treg from rats with transplant tolerance do not proliferate to specific-donor in the absence of key cytokines (26, 31). rIL-5 in culture enhanced their proliferation to specific-donor Lewis with a Stimulation Index that was signifiantly greater than that to self (p<0.01) and third-party PVG (p<0.001) (**Figure 6C** right panel). Proliferation to self or to third-party was not enhanced by addition of rIL-5. (**Figure 6C**, right panel).

CD4^+^CD25^+^ cells from sham-treated hosts, alone or with rIL-5 had no proliferation to specific donor, self or third party simulators (**Figure 6D**, middle panel). These animals had rejected their grafts and would not be expected to have activated Treg surviving 56 days post-transplant. Cultures of CD4^+^CD25^+^ cells from rats receiving long-term rIL-5, taken at day 60 post-transplantation, failed due to malfunction of an incubator and could not be repeated due to animal ethics issues.

### RT-PCR of mRNA for Transcription Factors, Cytokines and Cytokine Receptors on CD4^+^CD25^+^ Cells From F344 Rats With Lewis Heart Grafts

RT-PCR of mRNA from CD4^+^CD25^+^T cells from peripheral lymphoid tissue of heart grafted animals taken on day 16 at the end of treatment for 10 days with rIL-5 was performed. Controls were CD4^+^CD25^+^ cells from naïve F344 animals that had not been transplanted with a heart graft and had no treatment (**Figure 7A**). CD4^+^CD25^+^T cells from heart graft recipients treated with rIL-5 had significantly greater expression of *foxp3* (p<0.001)*, Irf4* (p<0.05), *Il5* (p<0.001), and higher *Il5Ra* (not significant) compared to CD4^+^CD25^+^T cells from naive F344 rats. These findings suggested that rIL-5 treatment activated Th2-like Treg, which expressed *Irf4* and *Il5.* There was also induction of Th1-like Treg markers *Ifngr (*p<0.05*), Il12rb2* (p,0.001*)* and *Ifng* (p<0.01), showing Th1-like Treg were also present.

CD4^+^CD25^+^ cells from rIL-5 and sham treated rats were also compared at around 60 days post transplantation (**Figure 7B**). CD4^+^CD25^+^ cells from long-term rIL-5 treated rats on day 60 post-transplantation, expressed more mRNA for *Foxp3* (p<0.01), *Irf4* (p<0.05) and *Il5* (p<0.01) than those from sham-treated rats with heart grafts, at 56 days post-transplantation. Sham treated rats did not receive rIL-5 and had rejected by day 31 post-transplant. The cells from short-term rIL-5 treated group (66 days post-transplantation), also had an increase in *Foxp3* (p<0.001), *Irf4* (p<0.01) and *il5* (p<0.001) compared to sham-treated at day 56 post-transplantation. Expression of Th1-like Treg marker *Ifng* (p<0.01) was also increased, but *Ifngr* and *Il12rb2* were not (**Figure 7B**).

## Discussion

Treatments to promote transplant tolerance could improve long-term allograft survival in patients. Immunoregulation is a complex process that involves a number of Treg pathways (23, 27, 43). In rodent models transplant tolerance is mediated by CD4^+^CD25^+^T cells (16, 17) that express Foxp3. During immune response to newly transplanted tissues, a variety of T effector cells are activated and can mediate rejection, including Th1 (44), Th2 (44, 45) and Th17 cells (46, 52, 55). Cytokines produced by these activated Th cells promote distinct pathways of activation of CD8^+^T cells, macrophages, and B cells. This study adds to a growing body of work showing they also activate distinct tTreg pathways (23, 28, 29, 43) to produce different subclasses of Treg, which contribute to tolerance to an allograft (43).

Resting/naïve tTreg are activated during all rejection responses, and can, if the graft is not totally destroyed, dominate rejection and allow recovery of graft function. In the model used, Lewis grafts in F344 recipients, one in ten grafts undergo transient rejection but fully recover and have good function long-term. This process is usually dominated by Type-1 activated Treg. In this study, rIL-5 treatment delayed rejection and allowed this natural regulatory process to dominate, permitting all grafts in rIL-5 treated host to survive without any immunosuppression. Thus, Type-2 activated Treg augmented the Type-1 activated Treg to inhibit the rejection response and promote tolerance to the allograft. In the short term rIL-5 treated the cessation of rIL-5 resulted in more rejection, however over time the grafts recovered and gained function similar to long term rIL-5 treated. We attributed this rejection to loss of IL-5 to promote Ts2 cells. The later recovery occurs if rejection is not total and is seen in a small proportion of F344 rats with Lewis heart grafts. We attribute this to induction of activated Treg by the grumbling rejection response.

Apart from delaying complete allograft rejection, less myocyte necrosis and mononuclear cells infiltration was identified in grafts in rIL-5 treated hosts. There was significantly fewer CD4^+^, CD8^+^, Foxp3^+^ infiltrating T cells in grafts of rIL-5 treated rats but macrophage infiltration was not reduced.

The best-defined pathway of activation of naïve/resting CD4^+^CD25^+^Foxp3^+^ tTreg involves Type-1 cytokines. This activation is a two-step process. In the first step, IL-2 activates naïve/resting tTreg, and in the presence of alloantigen generates donor-specific activated Treg (28, 29, 50, 51) that express receptors for Type-1 cytokines, including IL-12 and IFN-γ (28, 29, 51). We identified these Ts1 cells by using Type I cytokines to activate CD4^+^CD25^+^cells from a naïve host *in vitro (* 28, 29, 51). In the second step of activation the phenotype of Ts1 cells can be further modified by stimulation to specific donor alloantigen and IL-12 (28) in the absence of IL-2. This second step induces Th1-like Treg to express both Foxp3 and *Tbet*, also to produce *Ifng* but not *Il2*. These Th1-like Treg are much more potent at suppressing rejection than tTreg or Ts1 cells (28). Th1-like Treg markedly delay rejection of fully allogeneic heart grafts (28).

CD4^+^CD25^+^ cells from animals with transplant tolerance, which includes alloantigen specific Treg, do not proliferate to specific donor alloantigen *in vitro*, but they can proliferate to donor alloantigen if either IFN-γ or IL-12 are present in the culture medium (28, 29). Treatment with rIL-12 in some models can delay rejection of an allograft, and this effect requires IFN-γ (54). IFN-γ has been shown by others to promote expansion of antigen-specific Treg (56, 57). Thus, IL-12 and/or IFN-γ may promote induction of Th1-like Treg and promote tolerance. Both of these Type-1 cytokines have the potential to also promote Th1 responses and rejection, however.

Relevant to this study is the pathway of activation of naïve/resting CD4^+^CD25^+^ cells by antigen and Type-2 cytokines (**Figure 1**) (29, 30, 32, 43). In the first step, tTreg activated by alloantigen and IL-4, in the absence of rIL-2 in the culture, are induced to express mRNA for the receptor of the Type-2 cytokine IL-5, not the receptors for Type-1 cytokines IFN-γ and IL-12 (29). These activated tTreg we named Ts2 cells (29). In a second step, Ts2 cells further proliferate in the presence of IL-5 and specific alloantigen to become Th2-like Treg. Therapy with rIL-5 inhibits acute allograft rejection and induction of Th1 and Th17 responses (34) and promotes Ts2 cells (30, 32). IL-5 promotes survival of transplant tolerance transferring CD4^+^T cells, which die *ex vivo* without key cytokines, one of which is IL-5 (33). CD4^+^CD25^+^ cells from animals with transplant tolerance do not respond to specific donor alloantigen unless key cytokines such as IL-5 are present (31). In this study, CD4^+^CD25^+^ cells from rIL-5 treated, but not sham treated, hosts had a proliferative response to specific donor that was enhanced by rIL-5 in culture.

In this study, the Type-2 cytokine milieu did not inhibit induction of Ts1 and Th1-like Treg especially early on at day 16. Longer term, at around 60 days post-transplant where rIL-5 therapy was stopped at day 16 post-transplant, molecules associated with Th1-like Treg were also induced in CD4^+^CD25^+^Foxp3^+^Treg. Thus, Type 1 and Type 2 activated Treg can be activated in parallel and are not mutually exclusive.

For some time, Th2 responses were thought to promote transplant tolerance (44, 58).

Although therapy with rIL-4 (59, 60) or rIL-13 (61) delayed rejection, in other models rIL-4 promoted rejection (62–64). Further, in adoptive transfer studies allospecific Th2 cells mediate rejection (44, 45). Th2 cytokines are produced during normal rejection where there is induction of Th2 cells that contribute to normal allograft rejection responses (58, 65).

We concluded that the effects of rIL-5 in this study are attributed to its role in activation of Type-2 Treg. IL-5 is a cytokine produced by Th2 cells and some regulatory T cells including Tr1 and Ts1 cells (29, 66). IL-5 is produced long-term by Th2 cells, after the initial burst of IL-4 production diminishes. IL-5 acts by binding to a specific IL-5 receptor, IL-5Rα, which has limited expression. In man, IL-5Rα is mainly expressed on eosinophils, basophils and mast cells, and their progenitors (67). IL-5Rα is not expressed by human effector T cells including Th1, Th2, Th17 cells, nor APC, monocytes and macrophages (67). Over 30 years ago, IL-5 was reported to act with rIL-2 to induce cytotoxic T cells (68), but this finding has not been reproduced. Until we described IL-5Rα expression on IL-4 and antigen activated Treg and the capacity of IL-5 to promote their proliferation and expansion (21, 22), there was no solid evidence that IL-5 activated any T cells. We showed that human Treg activated by alloantigen and rIL-4 (not rIL-2) also are induced to express IL-5Rα (30)

IL-5 can activate CD5^+^B1 cells that express IL-5Rα (69) to produce natural IgM antibodies in response to bacterial stimulation (70, 71). IL-5 promotes murine, but not human, B cells to switch to produce non-complement fixing immunoglobulin isotypes IgG1 and IgE (67). rIL-5 therapy in autoimmunity does not induce a switch in Ig isotypes nor reduce Ig titres (30). IL-5, but not IL-4, induces expression of CD25 on activated B cells (72, 73) and leads to release of soluble CD25 (74), which could consume IL-2. The effect of IL-5 on B cells, as well as of anti-CD25 mAb on activated B cells, was not examined. We cannot exclude that B cells activation contributed to the rIL-5 effect on allograft rejection.

The results of this study are consistent with our findings in autoimmune models that rIL-5 therapy reduces immune inflammation (30, 32). The allograft model we used has only a class I MHC and multiple minor incompatibilities (38, 39, 75) making rejection slower than with both a class I and II MHC mismatch. In a neonatal heart transplant model, rIL-5 therapy delayed rejection and inhibited production of IFN-γ and IL-2.

Our findings of accelerated rejection by blocking IL-4 or by depleting CD25^+^cells are consistent with CD4^+^CD25^+^ Treg in the host being activated to Ts2 cells by alloantigen and the IL-4 produced by the alloantigen-activated effector T cells. In autoimmunity, blocking IL-4 and depleting CD25^+^ cells also abrogate the ability of rIL-5 to promote Ts2 cells to reduce immune injury (30, 32). In both autoimmunity and allograft rejection, the activation of Treg by Type-2 cytokines reduces inflammation.

In this study, we showed that Ts2 cells re-cultured with the same donor alloantigen and rIL-5 were induced to express mRNA for the Th2 transcription factors *Gata3* and *Irf4*, together with *il5.* Thus, *in vitro* we showed induction of Th2-like Treg. The CD4^+^CD25^+^T cells from rats with an allograft that had been treated with rIL-5 long-term had cells with a Th2-like Treg phenotype, in that they expressed *Foxp3, Irf4* and *Il5.* IRF4 is a transcription factor that is induced by TCR activation by antigen (76, 77) and the activation of a variety of immune cells in a Type 2 response (78) including antigen-activated Treg that control Th2 responses (36, 37). GATA-3 is the master transcription factor for Th2 responses. Further, in this study we showed *in vitro* induced Th2-like Treg expressed mRNA for *Il5*, which is not expressed by naïve Treg or Ts2 cells. The findings in this study were consistent with rIL-5 therapy promoting antigen-specific Treg that include Th2-like Treg.

In our studies in several models of alloimmunity (34) and autoimmunity (30, 32, 79), rIL-5 therapy was well tolerated. Mice with transgenes for IL-5 have high levels of IL-5 and eosinophilia, but remain healthy (38, 39, 80). High levels of IL-5 produced by Th2 responses to parasitic infections induces eosinophilia but has no adverse effects. The impairment of autoimmunity by parasitic infection in part depends on IL-5 and CD25^+^T cells activated by IL-4 (23).

CD4^+^CD25^+^cells from heart grafted animals treated with rIL-5 for 10 days, at the end of rIL-5 treatment, had increased proliferation to specific donor Lewis that was partially enhanced by addition of rIL-5 to cultures. These cells did not respond to self or third-party stimulator cells, even in the presence of rIL-5.

CD4^+^CD25^+^ cells of animals treated for 10 days with rIL-5, whose allografts survived >60 days, lacked reactivity to specific donor alloantigen unless rIL-5 was present in the cultures. These findings are consistent with an alloantigen-specific response of the tolerant CD4^+^CD25^+^T cells, we have recently described (31). Briefly, CD4^+^CD25^+^T cells from animals tolerant to a graft have no reactivity to donor antigen in the absence of cytokines such as IL-5 (31). Tolerant Type-2 cytokine activated Treg are dependent on IL-5, so cells have no response if rIL-5 is not present. Moreover, in the absence of rIL-5 *in vivo*, the alloantigen-specific Ts2 cells did not survive and other Treg, such as Ts1 and Th1-like Treg were activated. These results suggest that rIL-5 therapy may need to be given long-term to sustain the Ts2 cells and induce Th2-like Treg that express IRF4 and produce IL-5.

The mechanisms by which antigen specific Treg suppress rejection are not fully understood. They can enter the sites of inflammation in the graft, where they neutralize effector responses, including by production of adenosine by CD39 and CD73 expressed by activated Treg (81). Other less well understood mechanisms require direct Treg contact with effector cells that appear to involve Class II MHC on activated Treg and release of perforin and granzyme. Studies of the effector function of activated Treg are complicated by their dependence on specific antigen stimulation and cytokines such as IFN-γ (29, 31, 82) and IL-12 (28, 31) in the case of Type-1 activated Treg, or IL-5 in the case of Type 2 activated Treg (31, 33). Antigen-specific Treg die in culture without the cytokines required to support their survival (31, 83), and do not suppress proliferation of effector T cells *in vitro* (31, 84, 85). This is a distinct difference to naïve/resting tTreg which inhibit antigen presenting cells and reduce activation of naïve effector T cells (25). Given there is no assay for assessing suppression of activated alloantigen specific CD4^+^CD25^+^Foxp3^+^Treg *in vitro*, we were unable to assess their function *ex vivo*.

This study showed rIL-5 promoted induction of Treg that inhibited rejection to promote induction of tolerance. The inability of IL-5 to promote effector T cells makes it a better candidate for induction of tolerance than Type-1 cytokines IFN-γ (56, 57, 82, 86, 87) or IL-12 (28, 29) that promote Th1-like Treg but also promote Th1 responses and NK cells. Type-I induced and activated Treg were also generated in rIL-5 treated hosts demonstrating the pathways were complimentary and not mutually exclusive.

Although a variety of regulatory mechanisms have been described to promote transplant tolerance, the dominant regulatory mechanism in most models involves CD4^+^CD25^+^Foxp3^+^Treg (16, 17, 23, 31, 43). Human CD4^+^CD25^+^CD127^lo^Treg activated by rIL-4 and alloantigen express IL-5Rα (27). Thus, therapy with rIL-5 or an analogue may be of use to induce antigen-specific activated CD4^+^CD25^+^ Treg in man and suggests a new pathway to control ongoing rejection.

## Data Availability Statement

The original contributions presented in the study are included in the article/**Supplementary Material**. Further inquiries can be directed to the corresponding author.

## Ethics Statement

The animal study was reviewed and approved by University of New South Wales Animal Ethic Committee.

## Author Contributions

BH: Participated in research design, writing of paper, and data analysis. RH: Participated in conduct of research, writing of paper, and data analysis. GT: Participated in conduct of research, writing of paper, and data analysis. CR: Participated in conduct of research, writing of paper, and data analysis. PW: Participated in conduct of research, writing of paper, and data analysis. PR: Participated in conduct of research.CW: Participated in conduct of research. AS: Participated in research design, writing of paper, and data analysis. NV: Participated in research design, conduct of research; writing of paper, and data analysis. SH: Participated in research design, writing of paper, and data analysis. All authors contributed to the article and approved the submitted version.

## Funding

This study was supported by funding from South West Sydney Local Health District, The University of New South Wales, Sydney and anonymous donations.

## Conflict of Interest 

BH and SH hold patents related to production of antigen specific Treg and tests of tolerance related to this work.

The authors declare that the research was conducted in the absence of any commercial or financial relationship that could be construed as a potential conflict of interest.

## Publisher’s Note

All claims expressed in this article are solely those of the authors and do not necessarily represent those of their affiliated organizations, or those of the publisher, the editors and the reviewers. Any product that may be evaluated in this article, or claim that may be made by its manufacturer, is not guaranteed or endorsed by the publisher.

## Supplementary Material

Supplementary Figure 1 Photomicrographs (x40) of transplanted Lewis heart grafts in F344 recipients.

Supplementary Figure 2 Photomicrographs (x40) of transplanted Lewis heart grafts in F344 recipients. Comparison of grafts from sham and rIL-5 treated recipients. Immunoperoxidase staining with monoclonal antibodies to CD4, CD8, Foxp3 and ED1 (a macrophage marker). Quantitation of infiltrate shown in **Figure 4D**.

## Abbreviations

BSA, bovine serum albumin; HRP, horse radish peroxidase; Ipi, intra-peritoneal injection; IL-, interleukin; mAb, monoclonal antibody; PBS, phosphate buffered saline; r, recombinant; Treg, T regulatory cells.

## References

[1] Nankivell BJ Kuypers DR. Diagnosis and Prevention of Chronic Kidney Allograft Loss. Lancet (2011) 378(9800):1428–37. doi: 10.1016/S0140-6736(11)60699-5 22000139

[2] Wedel J Bruneau S Kochupurakkal N Boneschansker L Briscoe DM. Chronic Allograft Rejection: A Fresh Look. Curr Opin Organ Transpl (2015) 20(1):13–20. doi: 10.1097/MOT.0000000000000155. Review. PMC4461362 25563987

[3] Dhaliwal A Thohan V. Cardiac Allograft Vasculopathy: The Achilles’ Heel of Long-Term Survival After Cardiac Transplantation. Curr Atheroscler Rep (2006) 8(2):119–30. doi: 10.1007/s11883-006-0049-1 16510046

[4] Pober JS Janr-wit D Qin L Telledes G. Interacting Mechanisms in the Pathogenesis of Cardiac Allograft Vasculopathy. Arterioscler Thromb Vasc Biol (2014) 34(8):1609–14. doi: 10.1161/ATVBAHA.114.302818 PMC4324618 24903097

[5] Hall BM de Saxe I Dorsch SE. The Cellular Basis of Allograft Rejection *In Vivo.* III. Restoration of First-Set Rejection of Heart Grafts by T Helper Cells in Irradiated Rats. Transplantation (1983) 36(6):700–5. doi: 10.1097/00007890-198336060-00023 6362147

[6] Libby P Pober JS. Chronic Rejection. Immunity (2001) 14(4):387–97. doi: 10.1016/S1074-7613(01)00119-4 11336684

[7] Bedi DS Riella LV Tullius SG Chandraker A. Animal Models of Chronic Allograft Injury: Contributions and Limitations to Understanding the Mechanism of Long-Term Graft Dysfunction. Transplantation (2010) 90(9):935–44. doi: 10.1097/TP.0b013e3181efcfbc 20703180

[8] Halloran PF Pereira AB Chang J Matas A Picton M De Freitas D. Microarray Diagnosis of Antibody-Mediated Rejection in Kidney Transplant Biopsies: An International Prospective Study (INTERCOM). Am J Transplant (2013) 13(11):2865–74. doi: 10.1111/ajt.12465 24119109

[9] Merten S Chen JC Ha H Plain K Boyd RA Penny MJ. The Cellular Basis of Cardiac Allograft Rejection: VIII. Mechanisms Underlying Delayed Allograft Rejection in PVG C6-Deficient Rats. Transplantation (1998) 65(9):1152–8. doi: 10.1097/00007890-199805150-00002 9603160

[10] Mitchell RN. Graft Vascular Disease: Immune Response Meets the Vessel Wall. Annu Rev Pathol (2009) 4:19–47. doi: 10.1146/annurev.pathol.3.121806.151449 18717641

[11] Deng MC Bell S Huie P Pinto F Hunt SA Stinson EB. Cardiac Allograft Vascular Disease: Relationship to Microvascular Cell Surface Markers and Inflammatory Cell Phenotypes on Endomyocardial Biopsy. Circulation (1995) 81(6):1647–54. doi: 10.1161/01.CIR.91.6.1647 7882470

[12] Bishop GA Waugh JA Landers DV Krensky AM Hall BM. Microvascular Destruction in Renal Transplant Rejection. Transplantation (1989) 48(3):408–14. doi: 10.1097/00007890-198909000-00011 2476878

[13] Sakaguchi S. Naturally Arising Foxp3-Expressing CD25+CD4+ Regulatory T Cells in Immunological Tolerance to Self and Non-Self. Nat Immunol (2005) 6(4):345–52. doi: 10.1038/ni1178 15785760

[14] Thornton AM Shevach EM. Suppressor Effector Function of CD4+CD25+ Immunoregulatory T Cells Is Antigen Nonspecific. J Immunol (2000) 164(1):183–90. doi: 10.4049/jimmunol.164.1.183 10605010

[15] Hall BM Jelbart ME Gurley KE Dorsch SE. Specific Unresponsiveness in Rats With Prolonged Cardiac Allograft Survival After Treatment With Cyclosporine. Mediation of Specific Suppression by T Helper/Inducer Cells. J Exp Med (1985) 162(5):1683–94. doi: 10.1084/jem.162.5.1683 PMC2187930 2932519

[16] Hall BM Pearce NW Gurley KE Dorsch SE. Specific Unresponsiveness in Rats With Prolonged Cardiac Allograft Survival After Treatment With Cyclosporine. III. Further Characterization of the CD4^+^ Suppressor Cell and Its Mechanisms of Action. J Exp Med (1990) 171(1):141–57. doi: 10.1084/jem.171.1.141 PMC2187663 2136906

[17] Hall BM Plain KM Verma ND Tran GT Boyd R Robinson CM. Transfer of Allograft-Specific Tolerance Requires CD4^+^CD25+T Cells, But Not IL-4 or TGF-β and Cannot Induce Tolerance to Linked Antigens. Transplantation (2007) 83(8):1075–84. doi: 10.1097/01.tp.0000259553.66185.2f 17452898

[18] Miyara M Yoshioka Y Kitoh A Shima T Wing K Niwa A. Functional Delineation and Differentiation Dynamics of Human CD4^+^ T Cells Expressing the FoxP3 Transcription Factor. Immunity (2009) 30(6):899–911. doi: 10.1016/j.immuni.2009.03.019 19464196

[19] Giganti G Atif M Mohseni Y Mastronicola D Grageda N Povoleri GA. Treg Cell Therapy: How Cell Heterogeneity can Make the Difference. Eur J Immunol (2021) 51(1):39–55. doi: 10.1002/eji.201948131 33275279

[20] MacDonald KN Piret JM Levings MK. Methods to Manufacture Regulatory T Cells for Cell Therapy. Clin Exp Immunol (2019) 197(1):52–63. doi: 10.1111/cei.13297 30913302 PMC6591148

[21] Terry LV Oo YH. The Next Frontier of Regulatory T Cells: Promising Immunotherapy for Autoimmune Diseases and Organ Transplantations. Front Immunol (2020) 11:565518. doi: 10.3389/fimmu.2020.565518 33072105 PMC7538686

[22] Pilat N Sprent J. Treg Therapies Revisited: Tolerance Beyond Deletion. Front Immunol (2020) 11:622810. doi: 10.3389/fimmu.2020.622810 33633742 PMC7902070

[23] Hall BM. CD4^+^CD25+ T Regulatory Cells in Transplant Tolerance; 25 Years on. Transplantation (2016) 110(12):2533–47. doi: 10.1097/TP0000000000001436 27861285

[24] Bishop GA Ierino FL Sharland AF Hall BM Alexander SI Sandrin MS. Approaching the Promise of Operational Tolerance in Clinical Transplantation. Transplantation (2011) 91(10):1065–74. doi: 10.1097/TP.0b013e318215e742 21544029

[25] Nomura M Plain KM Verma N Robinson CM Boyd R Hodgkinson SJ. The Cellular Basis of Cardiac Allograft Rejection. IX. Ratio of Naive CD4^+^CD25^+^ T Cells/CD4^+^CD25- T Cells Determines Rejection or Tolerance. Transpl Immunol (2006) 15(4):311–8. doi: 10.1016/j.trim.2006.01.003 16635754

[26] Hall BM Robinson CM Plain KM Verma ND Carter N Boyd RA. Studies on Naïve CD4^+^CD25^+^T Cells Inhibition of Naïve CD4+CD25-T Cells in Mixed Lymphocyte Cultures. Transpl Immunol (2008) 18(4):291–300. doi: 10.1016/j.trim.2007.09.002 18158114

[27] Hall BM Verma ND Tran GT Hodgkinson SJ. Distinct Regulatory Cd4^+^T Cell Subsets; Differences Between Naïve and Antigen Specific T Regulatory Cells. Curr Opin Immunol (2011) 23:1–7. doi: 10.1016/j.coi.2011.07.012 21840184

[28] Verma ND Hall BM Plain KM Robinson CM Boyd R Tran GT. Interleukin-12 (IL-12p70) Promotes Induction of Highly Potent Th1-Like CD4^+^CD25^+^ T Regulatory Cells That Inhibit Allograft Rejection in Unmodified Recipients. Front Immunol (2014) 9:190. doi: 10.3389/fimmu.2014.00190 PMC4023029 24847323

[29] Verma ND Plain KM Nomura M Tran GT Robinson C. Cd4^+^Cd25^+^T Cells Alloactivated *Ex Vivo* by IL-2 or IL-4, Become Potent Alloantigen Specific Inhibitors of Rejection With Different Phenotypes, Suggesting Th1 and Th2 Responses Activate by Separate Pathways. Blood (2009) 113(2):479–87. doi: 10.1182/blood-2008-05-156612 18827184

[30] Tran GT Hodgkinson SJ Carter NM Verma ND Plain KM Boyd R. Interleukin-5 (IL-5) Promotes Induction of Antigen Specific CD4^+^CD25^+^T Regulatory Cells That Suppress Autoimmunity. Blood (2012) 119(19):4441–50. doi: 10.1182/blood-2011-12-396101 22310911

[31] Hall BM Robinson CM Plain KM Verma ND Tran GT Nomura M. Changes in Reactivity *In Vitro* of CD4+CD25+ and CD4^+^CD25- T Cell Subsets in Transplant Tolerance. Front Immunol (2017) 8:994. doi: 10.3389/fimmu.2017.00994 28878770 PMC5572370

[32] Tran GT Wilcox PL Dent LA Robinson CM Carter N Verma ND. Interleukin-5 Mediates Parasite-Induced Protection Against Experimental Autoimmune Encephalomyelitis and Is Associated With Induction of Antigen-Specific CD4^+^CD25^+^Treg. Front Immunol (2017) 8:1453. doi: 10.3389/fimmun.2017.01453 29163523 PMC5671975

[33] Hall BM Plain KM Tran GT Verma ND Robinson CM Nomura M. Cytokines Affecting CD4^+^T Regulatory Cells in Transplant Tolerance. Interleukin-5 (IL-5) Promotes Survival of Alloantigen Specific CD4+ T Regulatory Cells. Transplant Immunol (2017) 43-44:33–4. doi: 10.1016/j.trim.2017.1006.1003 28652007

[34] He XY Verma N Chen J Robinson C Boyd R Hall BM. IL-5 Prolongs Allograft Survival by Downregulating IL-2 and IFN-Gamma Cytokines. Transplant Proc (2001) 33(1-2):703–4. doi: 10.1016/S0041-1345(00)02212-0 11267027

[35] Tominaga N Ohkusu-Tsukada K Udono H Abe R Matsuyama T Yui Y. Development of Th1 and Not Th2 Immune Responses in Mice Lacking IFN-Regulatory Factor-4. Int Immunol (2003) 15(1):1–10. doi: 10.1093/intimm/dxg1001 12502720

[36] Zheng Y Chaudhry A Kas A deRoos A Kim P Chu JM. Regulatory T-Cell Suppressor Program Co-Opts Transcription Factor IRF4 to Control TH2 Responses. Nature (2009) 458:351–6. doi: 10.1038/nature07674. (319 March 2009). PMC2864791 19182775

[37] Huber M Lohoff M. IRF4 at the Crossroads of Effector T-Cell Fate Decision. Eur J Immunol (2014) 44(7):886–1895. doi: 10.1002/eji.201344279 24782159

[38] Russell ME Hancock WW Akalin E Wallace AF Glysing-Jensen T Willett TA. Chronic Cardiac Rejection in the LEW to F344 Rat Model. Blockade of CD28-B7 Costimulation by CTLA4Ig Modulates T Cell and Macrophage Activation and Attenuates Arteriosclerosis. J Clin Invest (1996) 97(3):833–8. doi: 10.1172/JCI118483 PMC507122 8609241

[39] Adams DH Russell ME Hancock WW Sayegh MH Wyner LR Karnovsky MJ. Chronic Rejection in Experimental Cardiac Transplantation: Studies in the Lewis-F344 Model. Immunol Rev (1993) 134:5–19. doi: 10.1111/j.1600-065X.1993.tb00637.x 8225375

[40] Ramirez F Stumbles P Puklavec M Mason D. Rat Interleukin-4 Assays. J Immunol Methods (1998) 221(1-2):141–50. doi: 10.1016/S0022-1759(98)00176-8 9894905

[41] Spicer ST Ha H Boyd RA He XY Carter N Tran G. IL-4 Therapy Prevents the Development of Proteinuria in Active Heymann Nephritis by Inhibition of Tc1 Cells. J Immunol (2001) 167(7):3725–33. doi: 10.4049/jimmunol.167.7.3725 11564788

[42] He XY Verma N Chen J Plain K Hall B. Cloning and Expression of Interleukin-5 From Rats. Transplant Proc (1999) 31(3):1574–6. doi: 10.1016/S0041-1345(99)00043-3 10331007

[43] Hall BM. T Cells: Soldiers and Spies-The Surveillance and Control of Effector T Cells by Regulatory T Cells. Clin J Am Soc Nephrol (2015) 10(11):2050–64. doi: 10.2215/CJN.06620714 PMC4633791 25876770

[44] Barbara JA Turvey SE Kingsley CI Spriewald BM Hara M Witzke O. Islet Allograft Rejection can be Mediated by CD4^+^, Alloantigen Experienced, Direct Pathway T Cells of Th1 and Th2 Cytokine Phenotype. Transplantation (2000) 70(11):1641–9. doi: 10.1097/00007890-200012150-00017 11152227

[45] Plain KM Verma ND Tran GT Nomura M Boyd R Robinson CM. Cytokines Affecting CD4^+^T Regulatory Cells in Transplant Tolerance. Interleukin-4 Does Not Maintain Alloantigen Specific CD4^+^CD25+Treg. Transplant Immunol (2013) 29:51–9. doi: 10.1016/j.trim.2013.1010.1003 24139939

[46] Itoh S Nakae S Axtell RC Velotta JB Kimura N Kajiware N. IL-17 Contributes to the Development of Chronic Rejection in a Murine Heart Transplant Model. J Clin Immunol (2010) 30(2):235–40. doi: 10.1007/s10875-009-9366-9 20130970

[47] Tellides G Dallman MJ Morris PJ. Mechanisms of Action of Interleukin-2 Receptor (IL_2R) Monoclonal Antibody (mAb) Therapy: Target Cell Depletion or Inhibition of Function? Transplant Proc (1989) 21(1 pt1):997–8. 2650306

[48] Tran GT Hodgkinson SJ Carter NM Killinsworth M Nomura M Verma ND. Membrane Attack Complex of Complement Is Not Essential for Immune Mediated Demyelination in Experimental Autoimmune Neuritis. J Neuroimmunol (2010) 229(1-2):98–106. doi: 10.1016/j.jneuroim.2010.07.010 20850187

[49] Tran GT Hodgkinson SJ Carter N Verma ND Robinson CM Plain KM. Autoantigen Specific IL-2 Activated CD4+CD25^+^T Regulatory Cells Inhibit Induction of Experimental Autoimmune Neuritis. J Neuroimmunol (2020) 341:577186. doi: 10.571016/j.jneuroim.572020.577186 32058174

[50] Tang Q Bluestone JA Kang SM. CD4^+^Foxp3^+^ Regulatory T Cell Therapy in Transplantation. J Mol Cell Biol (2012) 4(1):11–21. doi: 10.1093/jmcb/mjr047 22170955 PMC3695644

[51] Verma ND Robinson CM Carter N Wilcox P Tran GT Wang C. Recently Alloactivated CD4^+^CD8-Cd25^+^T Regulatory Cells Express CD8alpha and Are Potent Suppressor Cells That Can Promote Transplant Tolerance Induction. Front Immunol (2019) 10:2397. doi: 10.3389/fimmu.2019.02397 31681288 PMC6802415

[52] Mitchell P Afzali B Lombardi G Lechler R. The T Helper 17-Regulatory T Cell Axis in Transplant Rejection and Tolerance. Curr Opin Organ Transpl (2009) 14(4):326–31. doi: 10.1097/MOT.1090b1013e32832ce32888e 19448538

[53] Penny MJ Boyd RA Hall BM. Role of T Cells in the Mediation of Heymann Nephritis. Ii. Identification of Th1 and Cytotoxic Cells in Glomeruli. Kidney Int (1997) 51:1059–68. doi: 10.1038/ki.1997.148 9083271

[54] Verma ND Boyd R Robinson C Plain KM Tran GT Hall BM. Interleukin-12p70 Prolongs Allograft Survival by Induction of Interferon Gamma and Nitric Oxide Production. Transplantation (2006) 82(10):1324–33. doi: 10.1097/01.tp.0000239519.56358.c1 17130782

[55] Antonysamy MA Fanslow WC Fu F Li W Qian S Troutt AB. Evidence for a Role of IL-17 in Alloimmunity: A Novel IL-17 Antagonist Promotes Heart Graft Survival. Transplant Proc (1999) 31(1-2):93. doi: 10.1016/S0041-1345(98)01453-5 10083023

[56] Feng G Gao W Strom TB Oukkam M Francis SM Wood KJ. Exogenous IFN-Gamma *Ex Vivo* Shapes the Alloreactive T-Cell Repertoire by Inhibition of Th17 Responses and Generation of Functional Foxp3+ Regulatory T Cells. Eur J Immunol (2008) 38(9):2512–27. doi: 10.1002/eji.200838411 PMC2988413 18792404

[57] Feng G Wood KJ Bushell A. Interferon-Gamma Conditioning *Ex Vivo* Generates CD25^+^CD62L^+^Foxp3^+^ Regulatory T Cells That Prevent Allograft Rejection: Potential Avenues for Cellular Therapy. Transplantation (2008) 86:578–89. doi: 10.1097/TP.0b013e3181806a60 18724229

[58] Plain KM Chen J Merten S He XY Davidson C Hall BM. Induction of Specific Tolerance to Allografts in Rats by Therapy With Non-Mitogenic, Non Depleting Anti-CD3 Monoclonal Antibody; Association With Th2 Cytokines Not Anergy. Transplantation (1999) 67(4):605–13. doi: 10.1097/00007890-199902270-00020 10071035

[59] He XY Chen J Verma N Plain K Tran G Hall BM. Treatment With Interleukin-4 Prolongs Allogeneic Neonatal Heart Graft Survival by Inducing T Helper 2 Responses. Transplantation (1998) 65(9):1145–52. doi: 10.1097/00007890-199805150-00001 9603159

[60] Wang C Tay SS Tran GT Hodgkinson SJ Allen RD Hall BM. Donor IL-4-Treatment Induces Alternatively Activated Liver Macrophages and IDO-Expressing NK Cells and Promotes Rat Liver Allograft Acceptance. Transpl Immunol (2010) 22(3-4):172–8. doi: 10.1016/j.trim.2009.11.005 19944758

[61] Davidson C Verma ND Robinson CM Tran GT Hodgkinson SJ Hall BM. IL-13 Prolongs Allograft Survival: Association With Inhibition of Macrophage Cytokine Activation. Transpl Immunol (2007) 17(3):178–86. doi: 10.1016/j.trim.2006.09.035 17331844

[62] Wang C Cordoba SP McLeod DJ Tran GT Hodgkinson SJ Hall BM. Posttransplant Interleukin-4 Treatment Converts Rat Liver Allograft Tolerance to Rejection. Transplantation (2005) 79(9):1116–20. doi: 10.1097/01.TP.0000161249.20922.16 15880053

[63] Piccotti JR Chan SY VanBuskirk AM Eichwald EJ Bishop DK. Are Th2 Helper T Lymphocytes Beneficial, Deleterious, or Irrelevant in Promoting Allograft Survival. Transplantation (1997) 63(5):619–24. doi: 10.1097/00007890-199703150-00001 9075827

[64] Piccotti JR Chan SY Goodman RE Magram J Eichwald EJ Bishop DK. IL-12 Antagonism Induces T Helper 2 Responses, Yet Exacerbates Cardiac Allograft Rejection: Evidence Against a Dominant Protective Role for T Helper 2 Cytokines in Alloimmunity. J Immunol (1996) 157(5):1951–7. 8757314

[65] Plain KM Fava L Spinelli A He XY Chen J Boyd R. Induction of Tolerance With Nondepleting Anti-CD4 Monoclonal Antibodies Is Associated With Down Regulation of Th2 Cytokines. Transplantation (1997) 64:1559–67. doi: 10.1097/00007890-199712150-00009 9415556

[66] Sanderson CJ. Interleukin-5, Eosinophils and Disease. Blood (1992) 79(12):3101–9. doi: 10.1182/blood.V79.12.3101.bloodjournal79123101 1596561

[67] Takatsu K Takaki S Hitoshi Y. Interleukin-5 and Its Receptor System: Implications in the Immune System and Inflammation. Adv Immunol (1994) 57:145–90. doi: 10.1016/S0065-2776(08)60673-2 7872157

[68] Takatsu K Kikuchi Y Takahashi T Honjo T Matsumoto M Harada N. Interleukin 5, a T-Cell-Derived B-Cell Differentiation Factor Also Induces Cytotoxic T Lymphocytes. Proc Natl Acad Sci USA (1987) 84(12):4234–8. doi: 10.1073/pnas.84.12.4234 PMC305059 3495803

[69] Vaux DL Lalor PA Cory S Johnson GR. *In Vivo* Expression of Interleukin 5 Induces an Eosinophilia and Expanded Ly-1B Lineage Populations. Int Immunol (1990) 2(10):965–71. doi: 10.1093/intimm/2.10.965 2078522

[70] Bertolini JN Sanderson CJ Benson EM. Human Interleukin-5 Induces Staphylococcal A Cowan 1 Strain-Activated Human B Cells to Secrete IgM. Eur J Immunol (1993) 23(2):398–402. doi: 10.1002/eji.1830230215 8436175

[71] Huston MM JPM Mettes HJ GT Huston DP. Human B Cells Express IL-5 Receptor Messenger Ribonucleic Acid and Respond to IL-5 With Enhanced IgM Production After Mitogenic Stimulation With Moraxella Catarrhalis. J Immunol (1996) 156(4):1392–401. 8568239

[72] Loughnan MS Takatsu K Harada N Nossal GJ. T-Cell-Replacing Factor (Interleukin 5) Induces Expression of Interleukin 2 Receptors on Murine Splenic B Cells. Proc Natl Acad Sci USA (1987) 84(15):5399–403. doi: 10.1073/pnas.84.15.5399 PMC298863 3110787

[73] Poudrier J Owens T. The Acquisition of Cytokine Responsiveness by Murine B Cells: A Role for Antigen and IL-5 in the Induction of IL-2 Receptors. Immunology (1994) 81(3):373–80. PMC1422333 8206511

[74] Loughnan MS Sanderson CJ Nossal GJ. Soluble Interleukin 2 Receptors Are Released From the Cell Surface of Normal Murine B Lymphocytes Stimulated With Interleukin 5. Proc Natl Acad Sci USA (1988) 85((9):3115–9. doi: 10.1073/pnas.85.9.3115 PMC280154 3129727

[75] Adams DH Wyner LR Karnovsky MJ. Experimental Graft Arteriosclerosis. II. Immunocytochemical Analysis of Lesion Development. Transplantation (1993) 56(4):794–9. doi: 10.1097/00007890-199310000-00004 8212197

[76] Mittrücker H-W Matsuyama T Grossman A Kündig TM Potter J Shahinian A. Requirement for the Transcription Factor LSIRF/IRF4 for Mature B and T Lymphocyte Function. Science (1997) 275(5299den):540–3. doi: 10.1126/science.275.5299.540 8999800

[77] Li P Spolski R Liao W Leonard WJ. Complex Interactions of Transcription Factors in Mediating Cytokine Biology in T Cells. Immunol Rev (2014) 261(1):141–56. doi: 10.1111/imr.12199 PMC4174316 25123282

[78] Gao Y Nish SA Jiang R Hou L Licona-Limón P Weinstein JS. Control of T Helper 2 Responses by Transcription Factor IRF4-Dependent Dendritic Cells. Immunity (2013) 39(4):722–32. doi: 10.1016/j.immuni.2013.1008.1028 PMC4110745 24076050

[79] Traugott U Reinherz EL Raine CS. Multiple Sclerosis: Distribution of T Cell Subsets Within Active Chronic Lesions. Science (1983) 219(4582):308–10. doi: 10.1126/science.6217550 6217550

[80] Dent LA. Eosinophilai in Transgenic Mice Expressing IL-5. J Exp Med (1990) 172(5):1425–31. doi: 10.1084/jem.172.5.1425 PMC2188679 2230651

[81] Dwyer KM Deaglio S Gao W Friedman D Strom TB Robson SC. CD39 and Control of Cellular Immune Responses. Purinergic Signal (2007) 3(1-2):171–80. doi: 10.1007/s11302-006-9050-y PMC2096766 18404431

[82] Nomura M Hodgkinson SJ Tran GT Verma ND Robinson C Plain KM. Cytokines Affecting CD4^+^T Regulatory Cells in Transplant Tolerance. Interferon-Gamma (IFN-G) Promotes Survival of Alloantigen Specific CD4^+^T Regulatory Cells. Transplant Immunol (2017) 42(Jun):24–33. doi: 10.1016/j.trim.2017.1005.1002 28487237

[83] Pearce NW Spinelli A Gurley KE Hall BM. Specific Unresponsiveness in Rats With Prolonged Cardiac Allograft Survival After Treatment With Cyclosporin V. Dependence of the CD4^+^ Suppressor Cell on the Presence of Alloantigen and Cytokines, Including Interleukin-2. Transplantation (1993) 55(2):374–80. doi: 10.1097/00007890-199302000-00027 8434390

[84] Pearce NW Berger MF Gurley KE Spinelli A Hall BM. Specific Unresponsiveness in Rats With Prolonged Cardiac Allograft Survival After Treatment With Cyclosporine. VI. *In Vitro* Alloreactivity of T Cell Subsets From Rats With Long-Surviving Allografts. Transplantation (1993) 55(2):380–9. doi: 10.1097/00007890-199302000-00028 8434391

[85] Nicolls MR Aversa GG Pearce NW Spinelli A Berger MF Gurley KE. Induction of Long-Term Specific Tolerance to Allografts in Rats by Therapy With an Anti-CD3-Like Monoclonal Antibody. Transplantation (1993) 55(3):459–68. doi: 10.1097/00007890-199303000-00001 8456460

[86] Willenborg DO Fordham SA Staykova IM Ramshaw IA Cowden WB. Interferon-Gamma Is Critical to the Control of Murine Autoimmune Encephalomyelitis and Regulates Both in the Periphery and in the Target Tissue; a Possible Role for Nitric Oxide. J Immunol (1999) 163:5278–86. 10553050

[87] Feng G Wood KJ Bushell A. Regulatory T Cell Enrichment by IFN-G Conditioning. Methods Mol Biol (2011) 677:281–301. doi: 10.1007/978-1-60761-869-0_20 20941618

