# Supplementary figures and images for: Interleukin-5 (IL-5) Therapy Prevents Allograft Rejection by Promoting CD4+CD25+ Ts2 Regulatory Cells That Are Antigen-Specific and Express IL-5 Receptor
Source: Front Immunol. 2021 Nov 29;12:714838. doi: 10.3389/fimmu.2021.714838 (PMC8667344; doi:10.3389/fimmu.2021.714838)

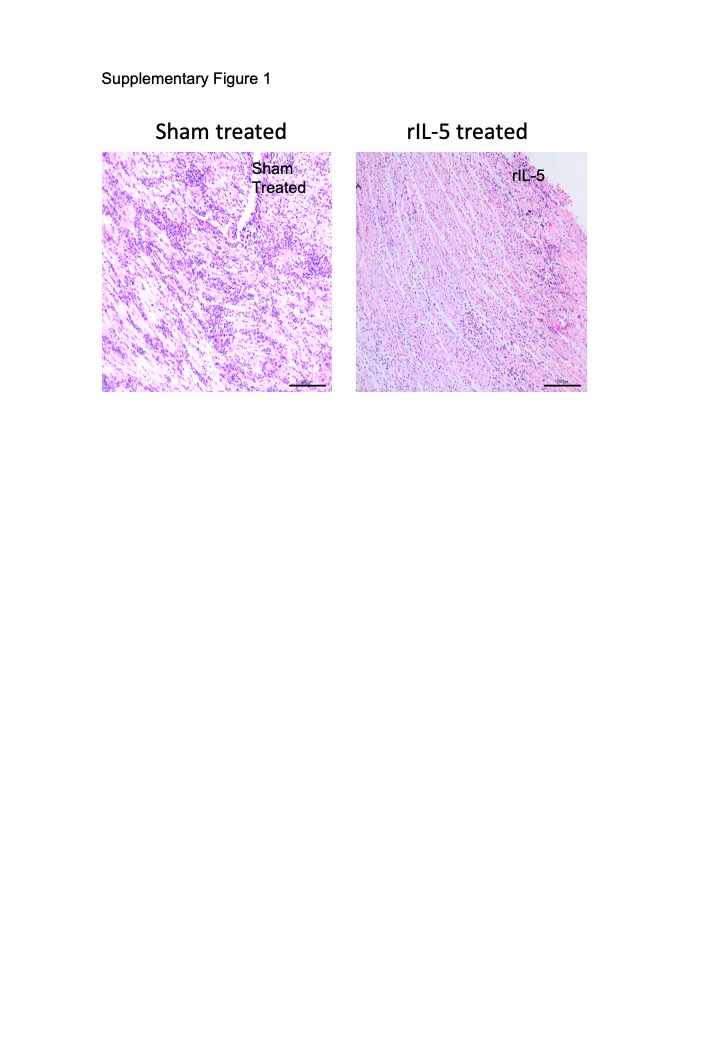

Supplement: Supplementary Figure 1 — Photomicrographs (x40) of transplanted Lewis heart grafts in F344 recipients. [file Image_1.jpg]

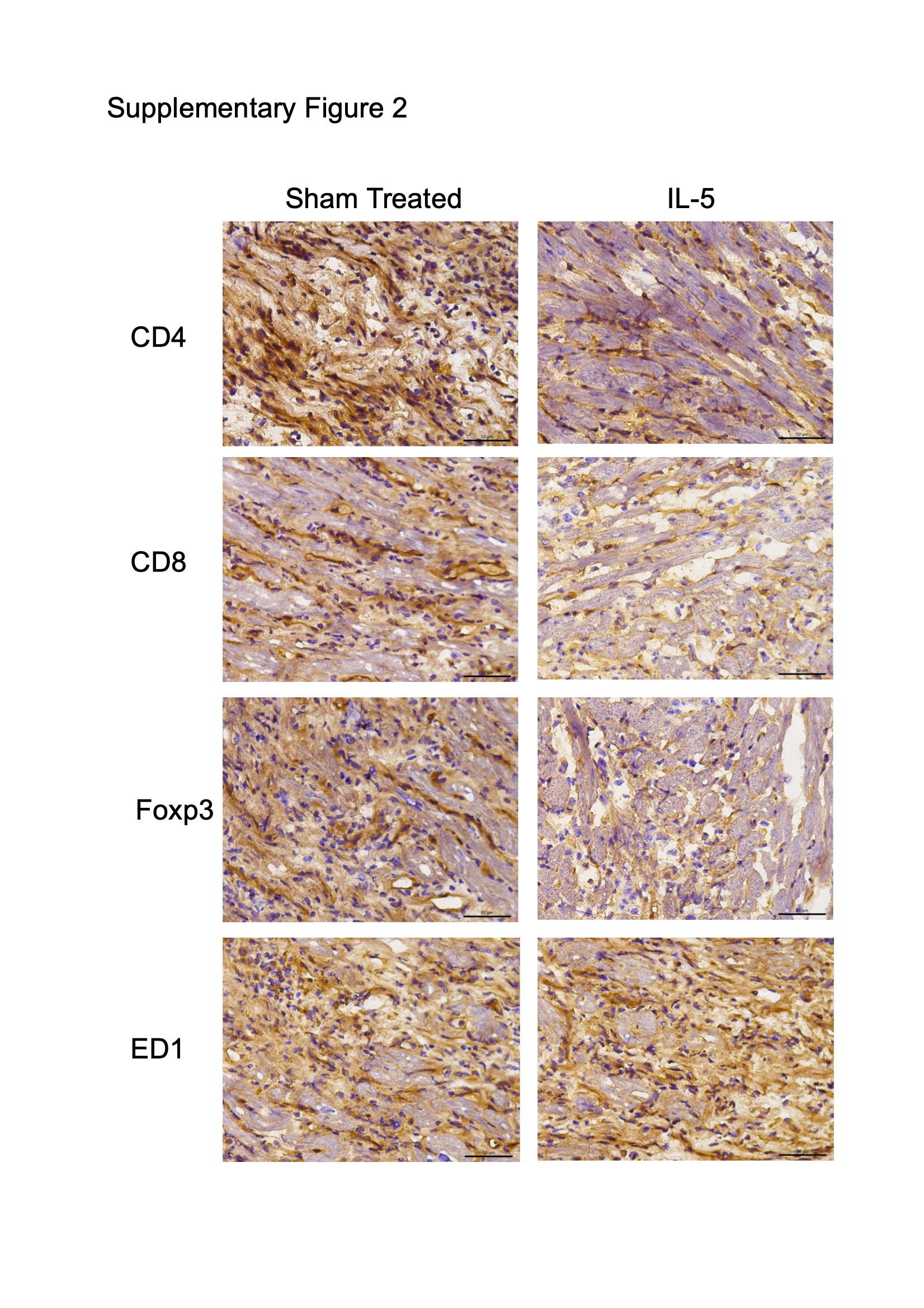

Supplement: Supplementary Figure 2 — Photomicrographs (x40) of transplanted Lewis heart grafts in F344 recipients. Comparison of grafts from sham and rIL-5 treated recipients. Immunoperoxidase staining with monoclonal antibodies to CD4, CD8, Foxp3 and ED1 (a macrophage marker). Quantitation of infiltrate shown in Figure 4D . [file Image_2.jpeg]
